# Every Tumour Counts: A Comprehensive Overview of Canine Oncology in Portugal

**DOI:** 10.3390/ani16010035

**Published:** 2025-12-23

**Authors:** Paula Brilhante-Simões, Ricardo Lopes, Leonor Delgado, Augusto Silva, Isabel Pires, Ricardo Marcos, Felisbina Queiroga, Justina Prada

**Affiliations:** 1 INNO Veterinary Laboratories, R. Cândido de Sousa 15, 4710-300 Braga, Portugal; paulabrilhante@inno.pt (P.B.-S.); leonordelgado@inno.pt (L.D.); augustosilva@inno.pt (A.S.); 2 Department of Veterinary and Animal Sciences, University Institute of Health Sciences (IUCS), CESPU, 4585-116 Gandra, Portugal; lopes.rmv@gmail.com; 3 Animal and Veterinary Research Centre (CECAV), Associate Laboratory for Animal and Veterinary Sciences (AL4AnimalS), University of Trás-os-Montes e Alto Douro (UTAD), 5000-801 Vila Real, Portugal; ipires@utad.pt (I.P.); fqueirog@utad.pt (F.Q.); 4 CEDIVET Veterinary Laboratories, Lionesa Business Hub, R. Lionesa 446 C24, 4465-671 Leça do Balio, Portugal; 5 Department of Veterinary Sciences, University of Trás–os–Montes e Alto Douro (UTAD), 5000-801 Vila Real, Portugal; 6 UNIPRO-Oral Pathology and Rehabilitation Research Unit, University Institute of Health Sciences-CESPU (IUCS-CESPU), 4585-116 Gandra, Portugal; 7 Cytology and Hematology Diagnostic Services, Laboratory of Histology and Embryology, Department of Microscopy, ICBAS-School of Medicine and Biomedical Sciences, University of Porto (U. Porto), Rua de Jorge Viterbo Ferreira, 228, 4050-313 Porto, Portugal; rmarcos@icbas.up.pt; 8 Animal Morphology and Toxicology Team, CIIMAR, Interdisciplinary Centre of Marine and Environmental Research, University of Porto (U. Porto), 4450-208 Matosinhos, Portugal

**Keywords:** cancer surveillance, epidemiology, histopathology, Portuguese dogs, veterinary pathology

## Abstract

Robust nationwide data on canine oncology in Portugal are scarce. We retrospectively reviewed 17,773 biopsy submissions received by a nationwide veterinary laboratory (2020–2024). After applying inclusion criteria, 6359 histopathology-confirmed tumours were analysed by behaviour, anatomical site, and host/geographical factors. Malignant and benign neoplasms occurred in near-equal proportions (50.4% vs. 49.6%). Tumour distribution was dominated by cutaneous/soft tissues (58.8%) and the mammary gland (24.1%), with all other sites individually rare. Dogs with malignant tumours were older than those with benign lesions (*p* < 0.001), and age differed by tumour behaviour and sex in two-way ANOVA (both *p* ≤ 0.001). Females accounted for 58.7% of submissions, and malignant disease was more frequent in females than in males (*p* < 0.001). Mixed-breed dogs formed the largest group; breed-specific logistic regression identified higher odds of malignancy in some pure breeds (e.g., Pug and American Staffordshire Terrier) and lower odds in others (e.g., Estrela Mountain Dog and Beagle). Multiplicity was recorded in a subset and was more common in females and in older dogs. Geographical analyses (NUTS2/NUTS3) showed, at most, weak regional variation and were not retained in multivariable models. These findings highlight the importance of early biopsy in new mass lesions, systematic mammary screening in bitches, breed-aware vigilance, and continued national surveillance to inform preventive care.

## Simple Summary

This five-year nationwide study analysed canine tumour biopsies submitted across Portugal. From 17,773 biopsy submissions, 6359 histopathology-confirmed neoplasms were included to describe tumour origin, frequency of malignancy and the types of dogs most affected. Almost four in five tumours were located in the skin/soft tissues (58.8%) or mammary gland (24.1%, predominantly in females), whereas neoplasms of the gastrointestinal tract, oral cavity, eyes, urinary system and other organs were comparatively uncommon. Dogs with malignant neoplasms were older than those with benign lesions. Mixed-breed dogs constituted the largest group; among purebreds, some breeds (e.g., Pug and American Staffordshire Terrier) had higher odds of malignancy, whereas others (e.g., Estrela Mountain Dog and Beagle) had lower odds. Multiplicity of tumours (subsequent tumours recorded over time) were more frequent in females and in older animals. Spatial analyses showed at most weak regional variation. For owners and clinicians, these findings support prompt assessment of any new mass, routine palpation of mammary chains in bitches and breed-aware vigilance, and they highlight the need for continued national surveillance to track trends and to guide prevention and care.

## 1. Introduction

Cancer represents one of the leading causes of death in canine populations [1,2,3,4,5,6,7,8]. In dogs, neoplasms not only compromise longevity but also have a significant impact on quality of life, and their treatment poses considerable clinical and ethical challenges for veterinary practitioners [9,10]. Because cancer is a major contributor to mortality, robust epidemiological data are needed [1,6,11,12,13,14,15,16,17,18,19,20,21]. Such data are essential to guide clinical decision-making, support early diagnostic strategies, and improve preventive measures [1,16,17,18,22,23,24,25,26,27,28,29,30,31,32,33]. From a One Health perspective, studies of cancer in dogs also provides insights into environmental risk factors, shared carcinogenic exposures, and comparative oncology that are relevant to both human and veterinary medicine [3,14,24,34,35,36,37,38,39].

Several international studies have investigated the epidemiology of canine neoplasms using different methodological approaches. Research from Europe [1,2,4,5,6,7,13,15,16,17,18,19,20,22,25,40,41,42,43,44,45,46], North America [10,21,47,48,49,50], South America [51,52], Asia [12,53,54,55], Africa [56] and Eurasia [57,58] has used data from veterinary cancer registries, insurance databases, and diagnostic laboratory submissions. These sources provided significant information regarding cancer incidence, anatomical distribution, and breed predispositions. Epidemiological studies have clarified the frequency of certain malignant neoplasms, such as mammary gland tumours [24,30,33,38,39,59,60,61,62,63,64,65], cutaneous tumours [11,55,66,67,68,69,70,71,72,73,74,75,76,77,78,79,80,81,82,83,84], oral tumours [66,77,78,82,84,85,86,87,88,89,90,91,92], lymphoma [93,94,95,96], osteosarcoma [97,98,99], haemangiosarcoma [100,101,102,103], and cancers of specific sites including lung [104,105], thyroid [106], gastrointestinal tract [107], and testis [108]. By focusing on specific cancer types or organ systems, these investigations have improved understanding of patterns of occurrence, disease risk and targeted health strategies.

In Portugal, publications on canine oncology remain scarce. Two studies based on the veterinary cancer registry Vet-OncoNet, describe and compare tumour frequency and characterise malignancy in dogs and cats [14,31]. Smaller-scale studies addressed specific tumour types, including cutaneous tumours [71,109], lymphoma [96,110], mast cell tumours [71,111], melanomas [112], and mammary tumours in a comparative study of dog-human context [39]. However, the national landscape of canine oncology is still only partially characterised. This is in clear contrast to several other European countries, where structured data collection has enabled long-term monitoring of cancer trends. Expanding and disseminating national data could substantially improve veterinary oncology practice in Portugal by supporting evidence-based approaches to diagnosis, treatment planning, and client education.

The present study addresses these gaps by providing new epidemiological evidence on canine neoplasms in Portugal. Using data from a veterinary diagnostic laboratory, it offers an overview of tumour distribution and frequency and highlights the most prevalent cancer types observed in Portuguese dogs.

## 2. Materials and Methods

### 2.1. Data Collection, Sampling and Diagnostic Procedures

A total of 6359 biopsy samples included in this study were submitted over a five-year period (2020–2024) to INNO Veterinary Laboratories (Braga, Portugal). These originated from 17,773 canine biopsy submissions, of which only histopathology-confirmed neoplastic lesions were retained for analysis. Samples were submitted by 371 veterinary practices distributed across all districts of mainland Portugal and the Autonomous Regions.

For each case, a standardised laboratory request form recorded key clinical metadata, breed, sex, age, clinical signs or presumptive diagnosis, anatomical sampling site, and requested analyses. Tissues were immersed in 10% neutral buffered formalin, processed by routine histopathological methods, and stained with haematoxylin and eosin (H&E). Diagnoses and nomenclature followed the World Health Organisation (WHO) classification of tumours of domestic animals [113]. Diagnoses were conducted/reviewed by two pathologists (LD and JP). Administrative and diagnostic data were retrieved from the Clinidata^®^ (version 5.3.25 Maxdata Software, S.A., Carregado, Portugal) and exported to Microsoft Excel^®^ (Microsoft, Redmond, WA, USA) for curation and analysis.

Age was stratified into seven categories according to previously described methodology [67,114]. Anatomical location was coded as: cutaneous and soft tissues; mammary gland (benign mammary tumour; malignant mammary tumour); oral cavity (tongue, lips, gingiva, palate, and mouth not otherwise specified—MNOS); gastrointestinal tract (stomach, intestine, liver, exocrine pancreas, gallbladder, and salivary glands); respiratory system (nasal cavity and lung); urinary system (kidney, ureters, bladder, and urethra); haemolymphatic system (lymph nodes, spleen, and thymus); musculoskeletal system (bone and joints); female reproductive system (uterus, ovaries and vagina); male reproductive system (penis, prostate and testis); ocular system; neuroendocrine system (endocrine pancreas, adrenal glands, thyroid gland, and aortic body); and heart.

Cases with non-neoplastic diagnoses were excluded from the analysis. For this study, a case was classified as multiple when additional material from the same animal was submitted at a later date than the index sample.

### 2.2. Statistical Analysis

Statistical evaluations were conducted using JMP^®^ version 14.3 (SAS Institute, Cary, NC, USA, 1989–2023), DATAtab^®^ (DATAtab e.U., Graz, Austria, 2024), and MedCalc^®^ Statistical Software version 20.006 (MedCalc Software Ltd., Ostend, Belgium). Proportional differences were assessed with chi-square (χ^2^) tests, with Fisher’s exact test applied when expected cell counts were <5. Age comparisons between benign and malignant tumours used the Mann–Whitney U test, and a two-way ANOVA evaluated age according to tumour behaviour (benign vs. malignant) and sex. Where relevant, effect size for contingency tables was expressed as Cramér’s V. To investigate breed-level associations with tumour malignancy, binary logistic regression models were fitted with tumour behaviour (benign vs. malignant) as the dependent variable and breed as a categorical predictor. Analyses were conducted both including and excluding mixed-breed dogs, with the latter performed to minimise heterogeneity. Results are presented as odds ratios (ORs) with 95% confidence intervals (CIs). Geographical associations (district) with tumour occurrence and behaviour were tested using chi-square test. Statistical significance was set at *p* ≤ 0.05.

## 3. Results

**Figure 1:**
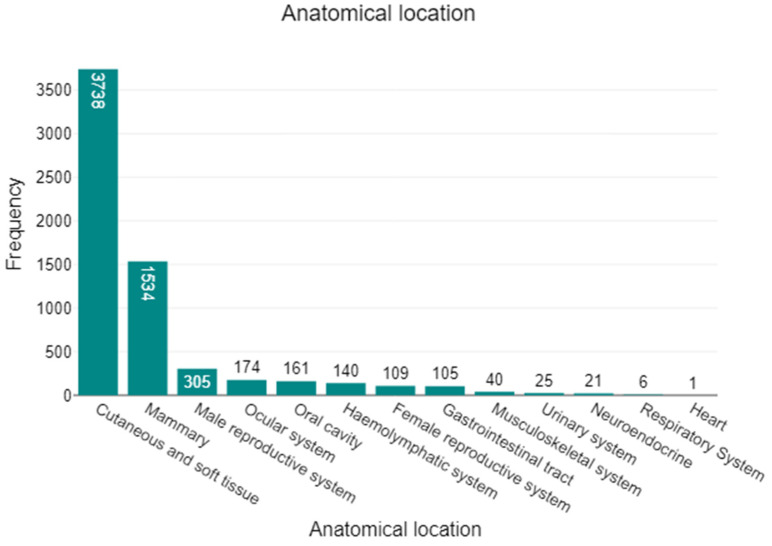
Anatomical distribution of all canine tumours (*n* = 6359). Tumour submissions were dominated by cutaneous and soft tissue (3738; 58.8%) and mammary (1534; 24.1%) sites, together accounting for 82.9% of cases. All other locations were comparatively infrequent: male reproductive system (305; 4.8%), ocular system (174; 2.7%), oral cavity (161; 2.5%), haemolymphatic tissues (140; 2.2%), female reproductive system (109; 1.7%), gastrointestinal tract (105; 1.7%), musculoskeletal system (40; 0.6%), urinary system (25; 0.4%), neuroendocrine (21; 0.3%), respiratory system (6; 0.1%), and body cavity (1; <0.1%).

Across the full cohort of 6359 histopathology submissions, tumours were predominantly located in the cutaneous and soft tissue (*n* = 3738; 58.8%) and mammary glands (*n* = 1534; 24.1%), together accounting for 82.9% of all cases. The remaining anatomical sites were far less represented: male reproductive system (*n* = 305; 4.8%), ocular system (*n* = 174; 2.7%), oral cavity (*n* = 161; 2.5%), haemolymphatic system (*n* = 140; 2.2%), female reproductive system (*n* = 109; 1.7%), gastrointestinal tract (*n* = 105; 1.7%), musculoskeletal system (*n* = 40; 0.6%), urinary system (*n* = 25; 0.4%), neuroendocrine (*n* = 21; 0.3%), respiratory system (*n* = 6; 0.1%), and heart (*n* = 1; <0.1%). This distribution is illustrated in Figure 1, which underscores the dominance of mammary and cutaneous/soft-tissue submissions within the dataset.

### 3.1. Diagnostic Yield

This retrospective study reviewed records from 6359 canine tumours subjected to histopathological evaluation. Of these, 3151 (49.6%, 95% CI: 48.3–50.8) were classified as benign and 3208 (50.4%, 95% CI: 49.2–51.7) as malignant, indicating an almost equal distribution between the two categories. All canine tumour samples are detailed in Supplementary Table S1, organised by anatomical site and diagnosis.

### 3.2. Age

Of the 6359 animals analysed, age data were available for 5868 (92.3%), whereas 491 (7.7%) requisition forms lacked this information and were excluded from age-based analyses. The age of included animals ranged from 2 months (≤1 years) to 23 years, with a median of 9 years (interquartile range [IQR]: 7–11). The distribution was as follows: 2.4% (95% CI: 1.6–3.2; *n* = 140) were ≤1 year, 1.5% (95% CI: 1.2–1.9; *n* = 90) were >1 to ≤2 years, 4.3% (95% CI: 3.8–4.9; *n* = 252) were >2 to ≤4 years, 22.4% (95% CI: 21.1–23.5; *n* = 1318) were >4 to ≤7 years, 38.4% (95% CI: 36.9–39.6; *n* = 2252) were >7 to ≤10 years, 29.8% (95% CI: 28.7–31.1; *n* = 1753) were >10 to ≤15 years, and 1.1% were ≥15 years (95% CI: 0.8–1.4; *n* = 63). The age distribution of the study population is summarised in Table 1.

**Figure 2:**
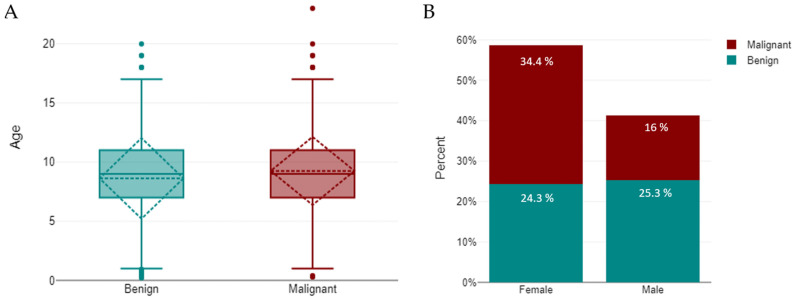
Distribution of benign and malignant tumours in dogs. Distribution of benign and malignant tumours in dogs. (**A**) Age distribution by tumour type, shown as boxplots indicating the median and interquartile range (IQR); whiskers denote minimum and maximum values, with outliers plotted individually. A significant difference was detected between benign and malignant groups (*p* < 0.001). (**B**) Sex distribution, illustrating the relative proportions of benign and malignant tumours in females and males.

Dogs with malignant tumours were slightly older than those with benign tumours (*p* < 0.001). The mean age of malignant cases was 9.26 ± 2.88 years (median = 9; *n* = 2964), compared with 8.62 ± 3.41 years (median = 9; *n* = 2904) for benign cases. A Mann–Whitney U-test confirmed this difference (U = 3,909,252; z = −6.11; *p* < 0.001; *r* = 0.08), indicating that malignant tumours tended to occur in older animals (Figure 2A). A Chi-square test further demonstrated that tumour behaviour was significantly associated with sex (χ^2^ = 242.6, *df* = 1, *p* < 0.001), with malignant tumours being more frequent in females (58.6%, 95% CI: 57.1–60.2; *n* = 2188/3731) than in males (38.8%, 95% CI: 37.0–40.7; *n* = 1020/2628) (Figure 2B).

Two-way ANOVA showed that age varied by tumour behaviour (benign vs. malignant; F = 59.0, *p* < 0.001, η^2^ = 0.01) and sex (F = 8.7, *p* = 0.003, η^2^ < 0.01), with a significant sex-behaviour interaction (F = 8.0, *p* = 0.005). Females were slightly older, and malignant cases occurred at older ages, especially in females (malignant: 9.41 ± 2.81 vs. 8.93 ± 3.00 years; benign: 8.53 ± 3.35 vs. 8.71 ± 3.47).

### 3.3. Sex

Overall, 3731 tumours (58.7%) occurred in female dogs and 2628 (41.3%) in males. In females, malignant tumours predominated (2188; 58.6%) over benign ones (1543; 41.4%), whereas in males the opposite pattern was observed, with benign tumours more frequent (1608; 61.2%) than malignant tumours (1020; 38.8%) (Figure 2B). A chi-square test confirmed a significant association between sex and tumour behaviour (χ^2^ = 242.6, *df* = 1, *p* < 0.001), although the effect size, measured by Cramér’s V (0.27), indicated a moderate association.

### 3.4. Breed

In total, 6359 dogs were included in the study, representing 99 breeds (98 pure breeds plus mixed breed). Mixed-breed dogs constituted the largest category (*n* = 2660; 41.8%), followed by Labrador Retriever (*n* = 729; 11.5%), French Bulldog (*n* = 295; 4.6%), Yorkshire Terrier (*n* = 274; 4.3%) and German Shepherd (*n* = 249; 3.9%). All other breeds individually accounted for <3% of cases.

Including mixed-breed dogs: Retaining mixed-breed dogs, the distribution of tumour behaviour was nearly balanced, with 1358 benign tumours (51.0%) and 1302 malignant tumours (49.0%), out of a total of 2660 cases. Across all 99 breeds (including mixed breed), the distribution of benign versus malignant tumours differed significantly by breed (χ^2^ = 144.29; *df* = 98; *p* = 0.002). Using mixed-breed as the reference in a logistic regression, several breeds had higher odds of malignancy, including Pug (*n* = 32; Odds Ratio (OR) = 3.73, 95% CI: 1.61–8.64; *p* = 0.002), American Staffordshire Terrier (*n* = 19; OR = 5.56, 95% CI: 1.62–19.14; *p* = 0.006), Boxer (*n* = 181; OR = 1.62, 95% CI: 1.19–2.20; *p* = 0.002), Poodle (*n* = 166; OR = 1.58, 95% CI: 1.15–2.18; *p* = 0.005), Teckel/Dachshund (*n* = 29; OR = 2.32, 95% CI: 1.05–5.11; *p* = 0.038), and Labrador Retriever (*n* = 729; OR = 95% CI: 1.27, 1.08–1.49; *p* = 0.004). In contrast, several breeds showed lower odds, including Estrela Mountain Dog (*n* = 49; OR = 0.46, 95% CI: 0.25–0.85; *p* = 0.013), Weimaraner (*n* = 20; OR = 0.35, 95% CI: 0.13–0.96; *p* = 0.041), Beagle (*n* = 136; OR = 0.69, 95% CI: 0.48–0.98; *p* = 0.037), Basset Hound (*n* = 34; OR = 0.43, 95% CI: 0.21–0.91; *p* = 0.027), and West Highland Terrier (*n* = 10; OR = 0.12, 95% CI: 0.01–0.92; *p* = 0.041). Table 2 summarises these data, presenting for each breed the number of cases, the proportion of malignant tumours with 95% confidence intervals, and the odds of malignancy relative to mixed-breed dogs (OR, 95% CI, *p*-value).

Excluding mixed-breed dogs: Given the heterogeneity of the mixed-breed category, breed-specific comparisons were also performed among purebreds only, where the distribution of tumour behaviour remained significantly different by breed (χ^2^ = 140.29; *df* = 97; *p* = 0.003).

To present effect sizes in a clinically interpretable way, we report malignant proportions with 95% Wilson confidence intervals for the most represented pure breeds (≥150 submissions): Labrador Retriever, 54.9% (95% CI: 51.2–58.4; *n* = 729); French Bulldog, 51.2% (95% CI: 45.5–56.8; *n* = 295); Yorkshire Terrier, 50.0% (95% CI: 44.1–55.9; *n* = 274); German Shepherd, 49.8% (95% CI: 43.6–56.0; *n* = 249); Boxer, 60.8% (95% CI: 53.5–67.6; *n* = 181); Golden Retriever, 53.6% (95% CI: 46.3–60.8; *n* = 179); Poodle, 60.2% (95% CI: 52.6–67.4; *n* = 166); Pinscher, 52.2% (95% CI: 44.5–59.7; *n* = 161); Cocker Spaniel, 44.7% (95% CI: 37.1–52.7; *n* = 152). For context, Beagle (just below the threshold) showed 39.7% (95% CI: 31.9–48.1; *n* = 136).

In a logistic regression restricted to purebred dogs, with the Labrador Retriever as the reference category, several breeds exhibited lower odds of malignancy, including Estrela Mountain Dog (*n* = 49; OR = 0.36, 95% CI: 0.19–0.68; *p* = 0.001), Siberian Husky (*n* = 22; OR = 0.38, 95% CI: 0.15–0.95; *p* = 0.039), Cocker Spaniel (*n* = 152; OR = 0.67, 95% CI: 0.47–0.95; *p* = 0.023), English Bulldog (*n* = 16; OR = 0.27, 95% CI: 0.09–0.86; *p* = 0.026), Border Collie (*n* = 17; OR = 0.34, 95% CI: 0.12–0.98; *p* = 0.046), Weimaraner (*n* = 20; OR = 0.27, 95% CI: 0.10–0.76; *p* = 0.013), Beagle (*n* = 136; OR = 0.54, 95% CI: 0.37–0.79; *p* = 0.001), West Highland Terrier (*n* = 10; OR = 0.09, 95% CI: 0.01–0.73; *p* = 0.024), and Shih Tzu (*n* = 44; OR = 0.52, 95% CI: 0.28–0.97; *p* = 0.039). In contrast, American Staffordshire Terrier (*n* = 19; OR = 4.39, 95% CI: 1.27–15.18; *p* = 0.020) and Pug (*n* = 32; OR = 2.94, 95% CI: 1.25–6.88; *p* = 0.013) showed higher odds of malignancy relative to Labradors (*n* = 729). Table 3 presents the number of cases, malignant tumour proportions with 95% confidence intervals, and logistic odds of malignancy (OR, 95% CI, *p*-value) for purebred dogs only, with the Labrador Retriever set as the reference category. Mixed-breed dogs were excluded from this analysis.

### 3.5. Anatomical Location

Cutaneous and soft tissues were the most frequently affected site (58.8%; 95% CI: 57.5–60.1; *n* = 3738), with benign tumours predominating (57.1%; 95% CI: 55.5–58.6). The mammary gland was the second most common location (24.1%; 95% CI: 22.9–25.4; *n* = 1534), where malignancy was markedly more frequent (80.1%; 95% CI: 77.9–82.2). The male reproductive system ranked third (4.8%; 95% CI: 4.3–5.3; *n* = 305) and was almost exclusively affected by benign lesions (99.7%; 95% CI: 98.1–100). The ocular system accounted for 2.7% (95% CI: 2.3–3.1; *n* = 174), with a strong predominance of benign tumours (96.6%; 95% CI: 92.6–98.5). Oral cavity tumours represented 2.5% (95% CI: 2.1–2.9; *n* = 161), the majority of which were benign (73.3%; 95% CI: 65.7–79.7). The female reproductive system contributed 1.7% of cases (95% CI: 1.4–2.0; *n* = 109), also with a predominance of benign lesions (80.7%; 95% CI: 72.4–87.0). The gastrointestinal tract comprised 1.7% of tumours (95% CI: 1.4–2.0; *n* = 105), the majority being malignant (79.1%; 95% CI: 70.0–86.0). Haemolymphatic tissues accounted for 2.2% (95% CI: 1.8–2.6; *n* = 140), with a predominance of malignant neoplasms (95.7%; 95% CI: 90.9–98.1). Less common locations included the urinary system (0.4%; 95% CI: 0.3–0.6; *n* = 25), neuroendocrine system (0.3%; 95% CI: 0.2–0.5; n = 21), musculoskeletal system (0.6%; 95% CI: 0.4–0.8; *n* = 40), respiratory system (0.1%; 95% CI: 0.0–0.2; *n* = 6), and heart (0.02%; 95% CI: 0.0–0.1; *n* = 1), all of which showed a clear predominance of malignancy (100% in the latter four categories). A chi-square test confirmed a strong association between anatomical location and tumour behaviour (χ^2^ = 1392.4; *df* = 12; *p* < 0.001). These results are detailed in Table 4.

Age and sex patterns varied by site. Mammary tumours occurred overwhelmingly in females (96.6% female; mean age 10.4 ± 3.5 years), with males rarely affected (3.4%; mean 10.2 ± 4.5 years). Cutaneous and soft tissue tumours showed near parity by sex (47.2% female; 52.9% male) and similar ages (females 9.7 ± 3.9; males 9.3 ± 3.8 years). Gastrointestinal tumours affected both sexes (45.3% female; 54.7% male) at ~10 years on average. Oral cavity tumours tended to present in older animals (female mean 12.3 ± 2.4; male mean 11.2 ± 3.9 years) and were more frequent in females (54.1%). Respiratory, urinary, and ocular tumours were male-biassed (respiratory 70.4% male; urinary 61.5% male; ocular 84.6% male), with urinary cases occurring at older ages (male mean 13.0 ± 1.6 years) and ocular cases in comparatively younger animals (male mean 7.2 ± 3.1 years). Haemolymphatic tumours also showed a male predominance (57.9%), whereas reproductive tumours were, as expected, largely female (95.2%). Descriptive statistics by site are presented in Table 5.

### 3.6. Multiple Neoplasia

Multiplicity data were available for a subset of cases. Of the 6359 tumours, multiplicity status was recorded for 3528 (55.5%), while 2831 (44.5%) lacked this information. Within the recorded subset, 73.8% (2603/3528) presented a subsequent neoplasm, 24.9% (877/3528) had two, 1.3% (45/3528) had three, and 0.1% (3/3528) had four lesions.

Among the 3528 dogs with multiplicity and sex recorded, nearly one-third of females (726/2253; 32.2%) presented multiple tumours, compared with approximately one in six males (199/1275; 15.6%). The association between sex and multiplicity was statistically significant (χ^2^ = 116.3, *df* = 1, *p* < 0.001; Cramér’s V = 0.18), indicating a small effect size. Most multiple lesions in females involved the mammary glands.

**Figure 3:**
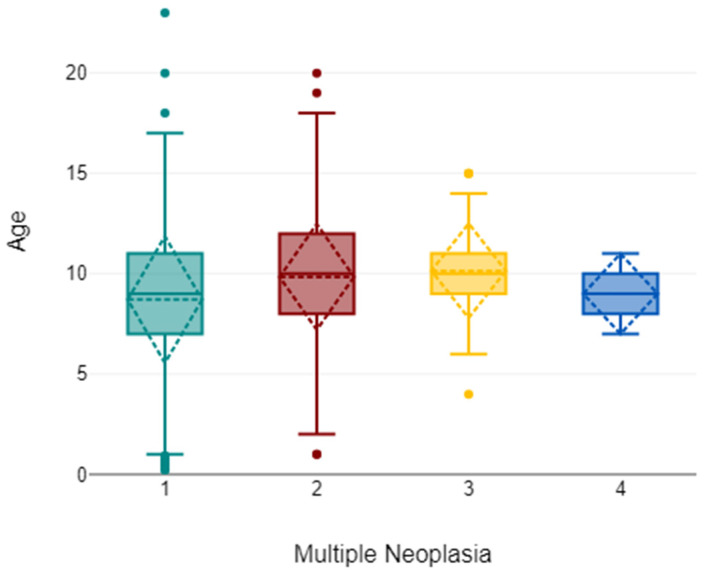
Age distribution according to tumour multiplicity (one to four lesions). Data are shown as boxplots displaying the median and interquartile range (IQR); whiskers represent the minimum and maximum values, with outliers plotted as individual points. Among dogs with age available (*n* = 3274/3528), median age increased from 9 years in animals with a single lesion to 10 years in those with two or three lesions, while the four-lesion group should be interpreted with caution given its very small size (*n* = 3). Age differences across multiplicity categories were statistically significant (χ^2^ = 85.31, *df* = 3, *p* < 0.001), with post hoc Dunn–Bonferroni tests confirming differences between one vs. two lesions and one vs. three lesions (adjusted *p* < 0.001 and *p* = 0.014, respectively).

In the subset with age available (*n* = 3274), age differed across multiplicity categories (χ^2^ = 85.31, *df* = 3, *p* < 0.001). The median age increased from 9 years in animals with a single lesion to 10 years in those with two or three lesions; post hoc Dunn–Bonferroni comparisons confirmed significant differences for one vs. two lesions and one vs. three lesions (adjusted *p* < 0.001 and *p* = 0.014, respectively). Comparisons involving the four-lesion group should be interpreted cautiously due to its very small size (Figure 3).

### 3.7. Geographical Location

To assess spatial heterogeneity, case counts were aggregated at two administrative levels (NUTS2 regions and NUTS3 sub-regions) and evaluated using Pearson’s χ^2^ tests of independence. A weak but statistically significant association between tumour behaviour (benign vs. malignant) and geography was observed at both scales (NUTS2: χ^2^ = 21.7, *df* = 8, *p* = 0.005; Pearson’s C = 0.08; NUTS3: χ^2^ = 263.43, *df* = 197, *p* = 0.001; C = 0.28). By contrast, tumour multiplicity (single vs. multiple lesions) showed no geographical structuring across NUTS2 regions (χ^2^ = 24.8, *df* = 24, *p* = 0.417; C = 0.10). Given the modest effect sizes and sparsity in some contingency cells, geographical variables were not retained as explanatory factors in subsequent analyses. The sampling frame comprised submissions from 371 veterinary practices distributed across the districts of mainland Portugal and the autonomous regions, supporting the generalisability of these spatial findings.

## 4. Discussion

Our nationwide review found an almost equal split between malignant and benign tumours in Portuguese dogs. This near parity aligns with data from other canine cancer registries [1,2,13,18,31,60]. For example, the Vet-OncoNet study (2019–2021) reported 46.2% of canine neoplasms as malignant [31], and early data from Denmark likewise showed comparable proportions of malignant (38%) and benign (45%) tumours [13]. By contrast, felines tend to present a much higher malignancy rate (nearly 79% in Vet-OncoNet), underscoring species differences in tumour biology and detection [31]. Cats develop fewer benign tumours, whereas dogs frequently develop benign growths (lipomas, adenomas, etc.) that are noticed and removed. This highlights a critical clinical point: in dogs, any new mass should be approached with caution, as the odds of malignancy are essentially as high as the odds of benignity. A lump should therefore not be assumed to be “just a fatty lump” without diagnostic confirmation.

### 4.1. Age as a Risk Factor

Age proved to be a significant factor in tumour behaviour. In our cohort, dogs with malignant neoplasms were significantly older on average than those with benign lesions. This pattern is consistently reported internationally. For instance, a large Italian registry noted median ages of 10 years for malignant tumours versus 9 years for benign [2]. Similarly, Vet-OncoNet found that malignant tumours present about 8 months later than benign on average [31]. The risk of malignancy rises steeply with age, Vet-OncoNet quantified a ~20% increase in odds of a tumour being malignant with each three-year increment in age. In Switzerland, overall tumour incidence peaks in the geriatric years (around 11 years old, the equivalent of ~1857 tumours per 100,000 dog-years) [1].

Biologically, these trends are unsurprising. Cancer is fundamentally an age-related disease, reflecting the accumulation of genetic mutations and epigenetic changes over time coupled with immunosenescence [1,2,6,8,23,25,28,50,115,116]. Dogs often remain free of malignant neoplasia in youth and mid-life, then experience a surge of cancers in their senior years once endogenous tumour-suppressing mechanisms wane [1,2,23,40,50,117].

From a clinical standpoint, this advocates for heightened vigilance as dogs age. Some authors have even suggested implementing cancer screening in middle-aged and senior dogs; for example, initiating routine checks around 7–8 years in mixed-breed dogs, and even earlier (before 6 years old) in breeds known to develop tumours at younger ages [2,40]. Systematic screening protocols in veterinary medicine are still evolving, our findings reinforce the importance of thorough annual health exams in older dogs. Early detection in this demographic could significantly improve outcomes, given that treatment of malignant tumours is more effective when they are small and localised.

### 4.2. Sex-Based Patterns

Our analysis revealed notable sex-related differences in canine oncology. Female dogs constituted a majority (58.7%) of submissions, and malignant disease was relatively more frequent in females than in males. This bias is corroborated by other studies and is largely attributable to the high incidence of mammary tumours in intact bitches [26,118,119,120]. In the Vet-OncoNet 2019–2021 data, female dogs had a significantly higher risk of malignancy than males (OR ≈ 1.19), whereas in cats no such sex effect was observed [31]. The Swiss Cancer Registry likewise found that female dogs had higher overall tumour incidence rates than males (850 vs. 679 per 100,000 dog-years) [1]. Historical data from Italy’s Genoa registry are even more striking: the overall cancer incidence in female dogs was nearly three times that of males, a difference explained almost entirely by the burden of mammary cancer in intact females [16].

In our study, mammary neoplasms accounted for roughly one-quarter of all tumours and the vast majority of those were in females. This underlines how a single cancer type heavily skews female cancer statistics. By contrast, males have no equivalent common tumour. Testicular tumours occur in males but are fewer in number and often benign. Other male-dominant tumours (e.g., perianal gland adenomas) do not rival the mammary tumour burden in frequency [1,11,17,18,23,26,43,118].

These observations carry important clinical implications. For intact female dogs, routine mammary-gland screening is crucial. The mammary chains should be systematically palpated at regular intervals (for instance, during annual check-ups), particularly once a bitch is beyond approximately 6–7 years of age, so that any developing mammary mass can be detected and addressed at the earliest opportunity [6,26,29,121]. Many mammary tumours in dogs can be cured with timely surgical removal when they are still small and well localised, ideally with the removal of the tributary lymph nodes [29,121].

In summary, sex hormones and related behaviours (pregnancy, lactation, oestrus cycles) play a significant role in canine oncologic epidemiology [122,123,124]. Notably, in species or populations where these influences are removed, the sex disparity diminishes. For example, in cats, where routine spaying of females is common, no significant sex difference in malignancy risk was observed [14,31]. This comparison further underscores that our female-biassed cancer patterns in dogs are not inevitable but rather are influenced by management decisions.

### 4.3. Breed-Specific Findings

Our nationwide data identified clear breed-specific differences in cancer occurrence and malignancy risk. Mixed-breed dogs formed the largest group in absolute numbers. However, when focusing on purebred dogs, certain breeds stood out at the extremes of malignancy odds. Pugs and American Staffordshire Terriers demonstrated significantly higher odds of their tumours were malignant (versus benign), whereas breeds like the Estrela Mountain Dog and Beagle showed the opposite trend, with malignancy under-represented relative to other breeds. These findings are consistent with, and expand upon, patterns reported in other studies [31].

The Vet-OncoNet study similarly found that breed was a significant determinant of malignancy risk in dogs, with breeds such as pit bulls (comparable to American Staffordshire Terriers dogs) and Boxers having higher malignancy propensities, while Yorkshire Terriers and Shih Tzus had notably lower malignancy risk [31]. A decade earlier, Denmark’s veterinary cancer registry had already noted a distinct breed predisposition: Boxers and Bernese Mountain Dogs showed a high risk of developing neoplasia (Standard Morbidity Ratios above 1), whereas German Shepherd Dogs and Danish/Swedish Farm Dogs had significantly lower risk than the average dog [13]. Likewise, an Italian population-based registry (Piedmont, 2001–2008) reported higher incidence rates in purebreds overall, particularly in small breeds like Yorkshire Terriers and in Boxers, with lower rates in some others [17]. Across these studies and ours, certain common themes emerge.

One recurring high-risk group is the brachycephalic and mastiff-type breeds (e.g., Boxers, Bulldogs, Pugs, Pit Bull/American Staffordshire Terriers). These breeds are frequently mentioned in the literature as cancer-prone [13,18,52,80,83,125]. Boxers, for instance, have long been known to suffer high rates of malignancies, especially mast cell tumours and certain brain tumours, which likely explains why so many of their tumours are malignant [13,18,52,71,80,83,125]. In our data, although Boxers were not explicitly highlighted (possibly due to a smaller sample or intermediate risk in our cohort), the finding that Pugs and American Staffordshire Terriers had high malignancy odds fits this pattern. Pugs are predisposed to mast cell tumours and malignant melanomas, and American Staffordshire Terriers/Pit Bulls also commonly develop high-grade mast cell tumours and other aggressive cancers [18,21,31,126]. These tumours can occur at relatively young ages in those breeds, further contributing to a high malignancy yield [2].

At the other end of the spectrum, breeds like Beagles and Estrela Mountain Dogs showing lower malignancy odds might indicate that these breeds more often develop benign tumours (for example, Beagles frequently get lipomas and benign skin adenomas), or it could reflect genuinely lower genetic predisposition to cancer. The Estrela Mountain Dog, a regional livestock guardian breed, might similarly benefit from a more diverse gene pool or historically less artificial selection, potentially reducing inherited cancer susceptibility. Another consideration is the “survivorship” bias: breeds prone to other fatal conditions (e.g., cardiac or orthopaedic issues) may not live long enough to develop as many cancers, thus appearing “protected” in cancer registries [127,128,129]. This could partly explain why some large breeds show lower cancer incidence in certain studies.

From a clinical perspective, these breed-associated patterns support the practice of breed-specific vigilance. Veterinary professionals should be aware of the particular cancer risks of breeds they commonly see. For high-risk breeds (Boxers, Golden Retrievers, Bernese Mountain Dogs, Rottweilers, flat-coated Retrievers, brachycephalic terriers, etc.), it is prudent to recommend more proactive monitoring, such as more frequent wellness checks or earlier diagnostics whenever clinical signs arise [1,18,31,115,126,130]. Owners of these breeds might be counselled to investigate even minor symptoms promptly (a small skin lump, a slight limp, etc.), given the elevated a priori risk of malignancy [6,28,50,130,131].

Conversely, for breeds with apparently lower cancer predisposition, one should not be complacent; they can and do get cancer, just perhaps at a lower rate or older age [1,13,74]. All dogs benefit from routine screening for tumours but knowledge breed risk refines the index of suspicion [1,2,28,50,132]. For now, the immediate recommendation is for clinicians to integrate breed information into their decision-making; much as human medicine considers family history as a risk factor, veterinary medicine can use breed as a proxy for genetic risk [35,37,133].

### 4.4. Tumour Topography Patterns

Our study confirms that the distribution of canine tumours by anatomical site is highly uneven, with certain locations overwhelmingly dominating the case mix. Nearly four in every five tumours in our series arose in just two anatomical systems: the integumentary (cutaneous and subcutaneous tissues) and the mammary gland. Approximately 59% of all tumours were of the skin or soft tissues, and roughly 24% were mammary tumours (virtually all in females), leaving all other organ systems to constitute only ~17% of cases combined. This pattern is very much in line with findings from other countries [6,11,18,19,134,135]. Skin neoplasms are consistently reported as the most common tumours in dogs [11,18,19,31,69,135]. For example, Denmark’s registry found 43% of canine neoplasms were cutaneous (not including soft tissue) and a further 28% involved the female reproductive system (primarily mammary tumours) [13]. The Swiss Cancer Registry (2008–2020) similarly noted that the skin was the top tumour location (34.6% of cases), followed by soft tissues (20.2%) and then the mammary glands (14.5%) [1]. When skin and subcutis are combined as many veterinary pathologists do, the Swiss data show ~55% of all tumours being dermal or subdermal, closely matching the ~59% we observed [1]. Italian registries have likewise reported skin and mammary neoplasms as the leading diagnoses in dogs [17].

Several factors likely contribute to this topographical distribution. Biologically, the skin (including associated glands and subcutaneous tissue) is exposed to various environmental factors and is a tissue with high cellular turnover, which may predispose it to neoplastic transformation [109,130,136,137,138]. Dogs also have numerous skin appendages (hair follicles, sebaceous glands, sweat glands) that can give rise to tumours. Mammary tissue, under hormonal influence in intact females, is similarly prone to tumour development, especially in dogs that experience many oestrous cycles [26,32,119,121].

However, a major reason for the predominance of cutaneous and mammary tumours in veterinary reports is that these are the tumours most readily detected by owners and most amenable to biopsy [26,139,140]. Owners commonly notice a lump on their dog’s skin or an enlarging mammary nodule and seek veterinary advice, leading to a surgical biopsy or excision that is submitted for pathology [139,140]. In contrast, neoplasms in internal organs (such as the lungs, liver, spleen, or inside the oral cavity) often go undetected until they cause serious clinical signs. Even then, not all owners opt for advanced diagnostics or surgical intervention, especially in older dogs [139,140]. Many internal tumours therefore remain unreported in pathology-based datasets. They may be diagnosed via imaging and palliated without biopsy, or the dog is euthanised due to clinical deterioration and no necropsy [1,16,18]. The Genoa Animal Tumour Registry explicitly noted that, because histopathology diagnosis was required for inclusion, cancers of internal organs (e.g., respiratory and gastrointestinal tract tumours) were likely underestimated in their data [16]. The same caveat applies to our findings: the low proportion of gastrointestinal or pulmonary tumours in our series does not mean such cancers are rare in Portugal; rather, it reflects that they are less often confirmed by biopsy in routine practice.

Even acknowledging these sampling biases, dogs do biologically develop far more skin and mammary tumours than many other types of cancer [11,18,31,69,71,135]. Primary lung tumours, for instance, are genuinely rare in dogs and are often overshadowed by metastatic cancers in the lung [141,142]. Certain cancers common in humans, such as colon carcinoma, are exceedingly uncommon in dogs [141,143]. Conversely, dogs far outstrip humans in the incidence of benign skin tumours (such as histiocytomas or lipomas) [1,18,144] and of mammary tumours (owing to the large population of intact females in dogs, whereas most women do not remain under constant oestrogen exposure) [39,145]. Thus, the “spectrum” of canine cancer differs from that of humans in ways that are both real and artefactual. In humans, the leading cancers by incidence are typically internal (e.g., lung, breast, colorectal, prostate). Although skin cancers are common, many registries exclude common non-melanoma skin cancers from reporting [37,146]. In dogs, if non-malignant skin tumours were similarly excluded, the data would shift, but even malignant skin tumours (mast cell tumours, soft-tissue sarcomas, melanomas) and malignant mammary tumours would still form a large share of the canine cancer burden [13,17,29,32].

Our geographic analysis found only weak regional variation in tumour type distribution, suggesting that across Portugal; no region had a markedly different profile of tumour types. This uniformity again points to host factors (age, sex, breed, reproductive status) as the primary drivers of these patterns, rather than local environmental factors creating different cancer spectra [2,11,18,26,31,88,117,120].

### 4.5. Clinical Implications and Recommendations for Veterinary Practice

Our findings support several concrete recommendations for veterinary clinicians. Foremost is the principle of maintaining a low threshold for diagnostic investigation of new masses. Given that roughly half of all lumps in dogs are malignant [1,2,13,31,60], the adage “when in doubt, cut it out” (or at least sample it) is well justified. Clinicians should encourage dog owners to report any new growth or swelling. Such masses should ideally be evaluated by cytology or histopathology rather than being assumed benign. The roughly equal prevalence of benign lesions should not discourage intervention; there is as much value in confirming a harmless lesion (and relieving owner anxiety) as in catching a malignant one early.

Another key implication is the importance of systematic mammary monitoring and control of reproductive risk factors. With one in four tumours in our study arising from the mammary chain, mostly in intact females, mammary neoplasia is clearly a predominant concern in countries where spaying is not universally practiced. We recommend that veterinarians discuss the benefits of spaying with owners of young female dogs not intended for breeding, highlighting the marked reduction in mammary cancer risk achieved by spaying before the first or second oestrus [121,147,148,149,150]. For those bitches that remain entire, vets should advise regular mammary exams at home (owners palpating their dogs’ mammary glands monthly) and perform thorough mammary palpation at every clinical visit.

Our breed-specific findings support breed-tailored counselling and surveillance. Breed is essentially the canine equivalent of “family history” in human medicine, encapsulating genetic predisposition [130]. For breeds known to carry elevated cancer risk (e.g., Boxer, Bernese Mountain Dog, Golden Retriever, Flat-Coated Retriever, Bulldog, etc.) [130], veterinarians might implement earlier or more frequent screening protocols. Clients with high-risk breeds should be made aware of the signs of common cancers in those breeds (for instance, explain to a Bernese owner the early signs of histiocytic sarcoma, or to a flat-coated retriever owner the risk of splenic tumours) [103].

Finally, our study highlights the value of ongoing cancer surveillance and record-keeping in veterinary medicine. We were able to reach these conclusions by aggregating data from thousands of cases over five years. Continued accumulation of such data will allow trends to be observed over time. We observed, for example, that geographical variation was minimal, but it would be useful to monitor whether any region or subpopulation shows a future increase in specific tumour types, which could indicate emerging risk factors. We consider it is important to continue developing the national canine tumour registry in Portugal, integrating as much data as possible and, ideally, supplementing it with detailed clinical information, similar to systems in other countries [1,17,135]. If linked with population data (dogs at risk), such registry would enable calculation of incidence rates and more robust epidemiological analyses.

This work intended to establish a foundation for more consistent national surveillance of canine oncology, and to support veterinary professionals in enhancing early detection and treatment. For practicing vets, contributing to and using such surveillance data can improve evidence-based decision-making. For example, if data show increasing mast cell tumour incidence in a breed or a reduction in mammary tumour incidence following spay campaigns, veterinarians can adjust their advice and resource allocation accordingly. In summary, the clinical message is to remain vigilant for cancer across all dogs, while using age, sex, and breed to inform the level of suspicion and preventive strategies. Our findings support a mindset of “screen early, biopsy readily, and educate owners”—an approach that can ultimately save canine lives and improve their quality of life, while giving owners confidence that cancer is not being overlooked. Ultimately, these efforts will contribute to improved animal health and welfare, while reinforcing the integration of veterinary oncology within a comparative and One Health framework.

### 4.6. One Health Perspective and Comparative Oncology

Our results not only inform veterinary practice but also have broader implications under the One Health framework, which recognises the interconnected health of humans, animals, and the environment. Dogs share our homes and many aspects of our environment, so the cancer patterns observed in dogs may reflect environmental carcinogenic exposures that also affect humans [6,15,17,59,132,138,151,152]. In our study, we did not find strong regional differences in canine cancer incidence or malignancy proportion; risk appeared fairly homogeneous across the country, and Vet-OncoNet similarly reported that district of residence was not a significant predictor of malignancy [31]. At this coarse spatial scale, there were no obvious “cancer hot-spots” in Portugal during the study period.

More granular analysis, however, can reveal overlaps between canine and human cancer patterns. A recent comparative epidemiological study in Northern Portugal examined canine mammary tumours alongside human breast cancer cases and found a striking spatial correlation: municipalities with higher incidence of female breast cancer in women also had higher incidence of mammary tumours in dogs, with a correlation coefficient of 0.85 [39]. Both were more frequent in urban than in rural areas [39]. This parallel suggests shared risk factors, such as greater exposure to pollution, sedentary lifestyles, or delayed childbearing in woman (and delayed spaying in bitches), all of which can increase breast/mammary cancer risk. It exemplifies how dogs can serve as sentinel species for environmental health [39,122,153,154]. Changes in canine cancer incidence may precede similar changes in humans, because dogs have shorter lifespans and may manifest effects of exposures sooner. Public health researchers have therefore advocated using pet dogs as sentinels to identify environmental carcinogens and hazards in communities [6,17,37,39,96]. By establishing baseline cancer occurrence in Portuguese dogs, provide a reference point to detect any future deviations that might warrant investigation.

### 4.7. Study Limitations

These findings should be interpreted in light of several limitations. This was a retrospective, submission-based series from a single diagnostic laboratory, which introduces selection and ascertainment bias. Readily detected, surgically accessible tumours (cutaneous/soft tissue, mammary) are likely over-represented, whereas internal neoplasms managed without histology are under-captured. The absence of a population denominator precludes incidence estimates and limits generalisability; temporal coverage (2020–2024) also restricts trend inference. Clinical metadata were incomplete (e.g., neuter status, hormonal history, treatments, outcomes), and age was missing in a minority of cases. Breed findings are tempered by small counts in many pure breeds, a large mixed-breed stratum, and potential confounding by age, sex and site. Geospatial signals were weak and based on aggregated counts without population-at-risk. Nonetheless, the large sample size, national catchment and standardised WHO nomenclature provide a robust cross-section of canine oncology in Portugal and a sound basis for future, prospective and registry-linked studies.

## 5. Conclusions

This nationwide, histopathology-based series provides one of the most comprehensive characterisations of canine tumours in Portugal to date. It shows a near-equal burden of malignant and benign disease and a marked predominance of cutaneous/soft-tissue and mammary sites. Dogs with malignant tumours were older than those with benign lesions, females contributed a larger share of cases and showed proportionally more malignancy—largely reflecting mammary neoplasia—and clear breed-level contrasts in malignancy odds were identified (elevated in Pug and American Staffordshire Terrier; reduced in Estrela Mountain Dog and Beagle). Tumour multiplicity was more frequent in females and increased with age, whereas geographical variation was weak, underscoring host factors as the main drivers of the observed patterns.

These findings establish a robust national baseline for canine oncology in Portugal and inform clinical practice by underscoring the importance of early cytology or biopsy of new masses, systematic mammary screening, discussion of prophylactic ovariohysterectomy in bitches, and breed-aware surveillance to expedite diagnosis in predisposed dogs, with the key implication that earlier detection and targeted prevention are both feasible and necessary.

## Figures and Tables

**Table 1:** Age group distribution of the study population with corresponding percentage, sample size (*n*), and 95% confidence intervals (CIs).

Age Group (Years)	% of Cases (*n*)	95% CI
≤1	2.4 (140)	1.6–3.2
>1–≤2	1.5 (90)	1.2–1.9
>2–≤4	4.3 (252)	3.8–4.9
>4–≤7	22.4 (1318)	21.1–23.5
>7–≤10	38.4 (2252)	36.9–39.6
>10–≤15	29.8 (1753)	28.7–31.1
≥15	1.1 (63)	0.8–1.4
Total	100 (5868)	–

CI, confidence interval.

**Table 2:** Breed-wise distribution of tumour behaviour with logistic odds of malignancy.

Breed	*n*	Benign (*n*, %)	Malignant (*n*, %)	OR for Malignancy (95% CI)	*p*-Value
Mixed-breed (reference)	2660	1358 (51.0%)	1302 (49.0%)	1.00 (reference)	–
American Staffordshire Terrier	19	3 (15.8%)	16 (84.2%)	5.56 (1.62–19.14)	0.006
Pug	32	7 (21.9%)	25 (78.1%)	3.73 (1.61–8.64)	0.002
Teckel/Dachshund	29	9 (31.0%)	20 (69.0%)	2.32 (1.05–5.11)	0.038
Boxer	181	71 (39.2%)	110 (60.8%)	1.62 (1.19–2.20)	0.002
Poodle	166	66 (39.8%)	100 (60.2%)	1.58 (1.15–2.18)	0.005
Labrador Retriever	729	329 (45.1%)	400 (54.9%)	1.27 (1.08–1.49)	0.004
Beagle	136	82 (60.3%)	54 (39.7%)	0.69 (0.48–0.98)	0.037
Estrela Mountain Dog	49	34 (69.4%)	15 (30.6%)	0.46 (0.25–0.85)	0.013
Basset Hound	34	24 (70.6%)	10 (29.4%)	0.43 (0.21–0.91)	0.027
Weimaraner	20	15 (75.0%)	5 (25.0%)	0.35 (0.13–0.96)	0.041
West Highland Terrier	10	9 (90.0%)	1 (10.0%)	0.12 (0.01–0.92)	0.041

CI, confidence interval; OR, odds ratio.

**Table 3:** Breed-wise distribution of tumour behaviour with logistic odds of malignancy (only purebred dogs).

	Breed	*n*	Benign (*n*, %)	Malignant (*n*, %)	OR for Malignancy (95% CI)	*p*-Value
	Labrador Retriever (reference)	729	329 (45.1%)	400 (54.9%)	1.00 (reference)	–
	American Staffordshire Terrier	19	3 (15.8%)	16 (84.2%)	4.39 (1.27–15.18)	0.020
	Pug	32	7 (21.9%)	25 (78.1%)	2.94 (1.25–6.88)	0.013
	Cocker Spaniel	152	84 (55.3%)	68 (44.7%)	0.67 (0.47–0.95)	0.023
	Beagle	136	82 (60.3%)	54 (39.7%)	0.54 (0.37–0.79)	0.001
	Shih Tzu	44	27 (61.4%)	17 (38.6%)	0.52 (0.28–0.97)	0.039
	Siberian Husky	22	15 (68.2%)	7 (31.8%)	0.38 (0.15–0.95)	0.039
	Estrela Mountain Dog	49	34 (69.4%)	15 (30.6%)	0.36 (0.19–0.68)	0.001
	Basset Hound	34	24 (70.6%)	10 (29.4%)	0.34 (0.16–0.73)	0.005
	Border Collie	17	12 (70.6%)	5 (29.4%)	0.34 (0.12–0.98)	0.046
	Weimaraner	20	15 (75.0%)	5 (25.0%)	0.27 (0.10–0.76)	0.013
English Bulldog		16	12 (75.0%)	4 (25.0%)	0.27 (0.09–0.86)	0.026
	West Highland Terrier	10	9 (90.0%)	1 (10.0%)	0.09 (0.01–0.73)	0.024

CI, confidence interval; OR, odds ratio.

**Table 4:** Distribution of benign and malignant canine tumours according to anatomical location.

Anatomic Location	Benign		Malignant		Total
	*n*	%	*n*	%	*n*
Cutaneous and soft tissue	2136	57.14%	1602	42.86%	3738
Mammary	305	19.88%	1229	80.12%	1534
Male reproductive system	304	99.67%	1	0.33%	305
Ocular system	168	96.55%	6	3.45%	174
Oral cavity	118	73.29%	43	26.71%	161
Haemolymphatic system	6	4.29%	134	95.71%	140
Female reproductive system	88	80.73%	21	19.27%	109
Gastrointestinal tract	22	20.95%	83	79.05%	105
Musculoskeletal system	0	0%	40	100%	40
Urinary system	4	16%	21	84%	25
Neuroendocrine system	0	0%	21	100%	21
Respiratory System	0	0%	6	100%	6
Heart	0	0%	1	100%	1
Total	3151	49.6%	3208	50.4%	6359

**Table 5:** Anatomical location: totals (*n*, %), main diagnoses (*n*, % of all tumours), sex distribution, and age descriptors.

Anatomical Location	Total (*n*; %)	Main Diagnoses (*n*; % of All Tumours)	Sex Distribution	Age (Years) ± SD
Cutaneous and soft tissue	3738 (58.8%)	Mast cell tumour (Grade II) (444; 6.98%) Perivascular wall tumour (313; 4.92%) Lipoma (311; 4.89%)	F: 1793 (47.9%) M: 1945 (52.0%)	F: 8.65 ± 3.34 M: 8.46 ± 3.39
Mammary	1534 (24.1%)	Malignant mammary tumour (1229; 19.33%) Benign mammary tumour (305; 4.80%)	F: 1501 (97.9%) M: 33 (2.1%)	F: 9.26 ± 2.63 M: 8.93 ± 2.67
Male reproductive system	305 (4.8%)	Interstitial (Leydig) cell tumour (126; 1.98%) Seminoma (79; 1.24%) Sertoli cell tumour (62; 0.97%)	M: 305 (100%)	M: 10.52 ± 2.50
Ocular system	174 (2.7%)	Meibomian adenoma (131; 2.06%) Meibomian epithelioma (28; 0.44%) Melanoma/Iridociliary adenoma (5; 0.08%)	F: 75 (43.1%) M: 99 (56.9%)	F: 9.72 ± 2.87 M: 9.12 ± 2.39
Oral cavity	161 (2.5%)	Peripheral odontogenic fibroma (94; 1.48%) Melanoma (37; 0.58%) Acanthomatous ameloblastoma (16; 0.25%)	F: 75 (46.6%) M: 86 (53.4%)	F: 9.57 ± 3.00 M: 8.94 ± 3.45
Haemolymphatic system	140 (2.2%)	Splenic haemangiosarcoma (63; 0.99%) Lymphoma (45; 0.71%) Splenic lymphoma (15; 0.24%)	F: 77 (55.0%) M: 63 (45.0%)	F: 10.45 ± 2.95 M: 9.19 ± 2.37
Female reproductive system	109 (1.7%)	Leiomyoma (49; 0.77%) Benign ovarian tumour (39; 0.61%) Ovarian carcinoma (15; 0.24%)	F: 109 (100%	F: 10.04 ± 3.10
Gastrointestinal tract	105 (1.7%)	Intestinal adenocarcinoma (27; 0.42%) Intestinal adenoma (17; 0.27%) Intestinal lymphoma (15; 0.24%)	F: 53 (50.5%) M: 52 (49.5%)	F: 9.34 ± 3.04 M: 9.22 ± 2.79
Musculoskeletal system	40 (0.6%)	Osteosarcoma (34; 0.53%) Chondrosarcoma (6; 0.09%)	F: 25 (62.5%) M: 15 (37.5%)	F: 9.52 ± 2.78 M: 8.14 ± 4.26
Urinary system	25 (0.4%)	Urothelial cell carcinoma (12; 0.19%) Renal adenocarcinoma (5; 0.08%) Renal haemangioma (1; 0.02%)	F: 9 (36.0%) M: 16 (64.0%)	F: 11.38 ± 5.04 M: 11.13 ± 3.10
Neuroendocrine system	21 (0.3%)	Thyroid carcinoma (13; 0.20%) Chemodectoma (3; 0.05%) Pheochromocytoma (2; 0.03%)	F: 10 (47.6%) M: 11 (52.4%)	F: 10.60 ± 2.63 M: 10.11 ± 1.45
Respiratory system	6 (0.1%)	Pulmonary adenocarcinoma (3; 0.05%) Nasal adenocarcinoma (2; 0.02%) Nasal transitional cell carcinoma (1; 0.02%)	F: 4 (66.7%) M: 2 (33.3%)	F: 12.00 ± 3.37 M: 8.00 ± 1.41
Heart	1 (<0.1%)	Cardiac haemangiosarcoma (1; 0.02%)	F: 0 (0.0%) M: 1 (100.0%)	M: 9.0 (single case)

The distribution of canine tumours by breed, anatomical location, and diagnosis is presented in Supplementary Table S2.

## Data Availability

The data presented in this study are available on request from the corresponding authors.

## Supplementary Materials

The following supporting information can be downloaded at https://www.mdpi.com/article/10.3390/ani16010035/s1, Supplementary Table S1: Canine tumours by anatomical location and diagnosis: totals (*n*, % of all cases) and within-location diagnosis (*n*, %); Supplementary Table S2: Distribution of canine tumours by breed, anatomical location, and diagnosis: totals (*n*; % of all cases) and within-location diagnosis (*n*; %).
