# Supplementary material for: Every Tumour Counts: A Comprehensive Overview of Canine Oncology in Portugal
Source: Animals (Basel). 2025 Dec 23;16(1):35. doi: 10.3390/ani16010035 (PMC12784951; doi:10.3390/ani16010035)
Supplement: Supplementary file 1 [file animals-16-00035-s001.zip › Table S1-dogs.pdf]

Supplementary Table S1. Canine tumours by anatomical location and diagnosis: totals (*n*, % of all cases) and within-location diagnosis (*n*, %).

Anatomical Location	Total (*n*; %)	Within-location Diagnosis (*n*; %)
Cutaneous and Soft tissue	3,738 (58.78%)	Mast cell tumour grade II (444; 11.88%)
		Perivascular wall tumour (313; 8.37%)
		Lipoma (311; 8.32%)
		Histiocytoma (264; 7.06%)
		Hepatoid adenoma (170; 4.55%)
		Haemangiosarcoma (150; 4.01%)
		Mast cell tumour grade I (151; 4.04%)
		Mast cell tumour grade III (140; 3.75%)
		Melanoma (124; 3.32%)
		Subcutaneous mast cell tumour (119; 3.18%)
		Haemangioma (118; 3.16%)
		Squamous cell carcinoma (118; 3.16%)
		Fibrosarcoma (112; 3.00%)
		Infundibular keratinizing acanthoma (107; 2.86%)
		Trichoepithelioma (105; 2.81%)
		Trichoblastoma (99; 2.65%)
		Melanocytoma (89; 2.38%)
		Squamous papilloma (78; 2.09%)
		Sebaceous epithelioma (76; 2.03%)
		Sebaceous adenoma (71; 1.90%)
		Hepatoid epithelioma (62; 1.66%)
		Plasmacytoma (53; 1.42%)
		Fibroma (45; 1.20%)
		Apocrine adenoma (44; 1.18%)
		Pilomatricoma (42; 1.12%)
		Apocrine adenocarcinoma (38; 1.02%)
		Schwannoma (44; 1.18%)
		Haemangiosarcoma (32; 0.86%)
		Myxosarcoma (25; 0.67%)
		Lymphoma (18; 0.48%)
		Anal sac adenocarcinoma (18; 0.48%)
		Matrical carcinoma (14; 0.37%)
		Apocrine ductal adenocarcinoma (14; 0.37%)
		Malignant trichoepithelioma (13; 0.35%)
		Apocrine ductal adenoma (10; 0.27%)
		Basal cell carcinoma (8; 0.21%)
		Ceruminous adenocarcinoma (8; 0.21%)
		Trichilemmoma (7; 0.19%)
		Liposarcoma (7; 0.19%)
		Basosquamous carcinoma (7; 0.19%)
		Hepatoid adenocarcinoma (6; 0.16%)
		Malignant plasmacytoma (8; 0.21%)
		Histiocytic sarcoma (5; 0.13%)
		Inverted squamous papilloma (5; 0.13%)
		Basal cell tumour (5; 0.13%)
		Extraskeletal chondrosarcoma (4; 0.11%)
		Cutaneous histiocytosis (4; 0.11%)
		Myxoma (4; 0.11%)
		Ceruminous adenoma (4; 0.11%)
		Lymphangioma (3; 0.08%)
		Extraskeletal osteosarcoma (3; 0.08%)
		Malignant schwannoma (3; 0.08%)
		Sebaceous adenocarcinoma (2; 0.05%)

		Clear cell adnexal carcinoma (2; 0.05%)<br>Liposarcoma (2; 0.05%)<br>Leiomyoma (1; 0.03%)<br>Merkel cell tumour (1; 0.03%)<br>Ductal adenocarcinoma (1; 0.03%)<br>Hepatoid carcinoma (1; 0.03%)<br>Lymphangiosarcoma (1; 0.03%)<br>Ganglioneuroma (1; 0.03%)<br>Leiomyosarcoma (1; 0.03%)<br>Sebaceous ductal adenoma (1; 0.03%)<br>Extraskeletal chondroma (1; 0.03%)<br>Neuroblastoma (1; 0.03%)
Mammary	1,534 (24.12%)	Complex carcinoma (541; 35.27%)<br>Tubulopapillary carcinoma (256; 16.69%)<br>Complex adenoma (133; 8.67%)<br>Benign mixed tumour (89; 5.80%)<br>Tubular carcinoma (83; 5.41%)<br>Carcinoma-and-malignant myoepithelioma (67; 4.37%)<br>Ductal carcinoma (50; 3.26%)<br>Adenoma – simple (50; 3.26%)<br>Carcinoma arising in a benign mixed tumour (46; 3.00%)<br>Intraductal papillary carcinoma (40; 2.61%)<br>Carcinosarcoma (28; 1.83%)<br>Solid carcinoma (24; 1.56%)<br>Ductal adenoma (13; 0.85%)<br>Comedocarcinoma (12; 0.78%)<br>Adenosquamous carcinoma (11; 0.72%)<br>Malignant myoepithelioma (10; 0.65%)<br>Osteosarcoma (9; 0.59%)<br>Anaplastic carcinoma (9; 0.59%)<br>Mixed carcinoma (9; 0.59%)<br>Mucinous carcinoma (8; 0.52%)<br>Spindle cell carcinoma (7; 0.46%)<br>Adenoma – simple (7; 0.46%)<br>Invasive micropapillary carcinoma (6; 0.39%)<br>Intraductal papillary adenoma (6; 0.39%)<br>Fibroadenoma (5; 0.33%)<br>Fibrosarcoma (4; 0.26%)<br>Chondrosarcoma (3; 0.20%)<br>Inflammatory mammary carcinoma (2; 0.13%)<br>Squamous cell carcinoma (1; 0.07%)<br>Fibroadenoma (1; 0.07%)<br>Lipoma (1; 0.07%)<br>Liposarcoma (1; 0.07%)<br>Cribriform carcinoma (1; 0.07%)<br>Lipid-rich carcinoma (1; 0.07%)
Male reproductive system	305 (4.80%)	Interstitial (Leydig) cell tumour (126; 41.31%)<br>Seminoma (79; 25.91%)<br>Sertoli cell tumour (62; 20.33%)<br>Mixed germ cell sex-cord stromal tumour (37; 12.13%)<br>Transmissible venereal tumour (1; 0.33%)
Ocular system	174 (2.74%)	Meibomian adenoma (131; 75.29%)<br>Meibomian epithelioma (28; 16.09%)<br>Melanoma (5; 2.87%)<br>Iridociliary adenoma (5; 2.87%)<br>Melanocytoma (4; 2.30%)<br>Iridociliary adenocarcinoma (1; 0.57%)

Oral cavity	161 (2.53%)	Peripheral odontogenic fibroma (94; 58.39%)<br>Melanoma (37; 22.98%)<br>Acanthomatous ameloblastoma (16; 9.94%)<br>Fibrosarcoma (4; 2.48%)<br>Granular cell myoblastoma (4; 2.48%)<br>Plasmacytoma (2; 1.24%)<br>Ameloblastic fibroma (2; 1.24%)<br>Malignant plasmacytoma (1; 0.62%)<br>Ameloblastic carcinoma (1; 0.62%)
Haemolymphatic system	140 (2.20%)	Splenic hemangiosarcoma (63; 45.00%)<br>Lymphoma (45; 32.14%)<br>Splenic lymphoma (15; 10.71%)<br>Splenic fibrosarcoma (6; 4.29%)<br>Splenic myelolipoma (5; 3.57%)<br>Splenic anaplastic sarcoma (3; 2.14%)<br>Splenic plasmacytoma (1; 0.71%)<br>Thymic lymphoma (1; 0.71%)<br>Splenic leiomyosarcoma (1; 0.71%)
Female reproductive system	109 (1.71%)	Leiomyoma (49; 44.96%)<br>Granulosa cell tumour (19; 17.43%)<br>Ovarian carcinoma (15; 13.76%)<br>Ovarian papillary adenoma (15; 13.76%)<br>Dysgerminoma (5; 4.59%)<br>Ovarian cystadenoma (2; 1.83%)<br>Ovarian surface adenoma (1; 0.92%)<br>Teca cell tumour (1; 0.92%)<br>Teratoma (1; 0.92%)<br>Endometrial carcinoma (1; 0.92%)
Gastrointestinal tract	105 (1.65%)	Tubular adenocarcinoma (17; 16.19%)<br>Intestinal lymphoma (15; 14.29%)<br>Hepatocellular carcinoma (12; 11.43%)<br>Villous adenoma (12; 11.43%)<br>GIST (9; 8.57%)<br>Hepatic haemangiosarcoma (7; 6.67%)<br>Mucinous adenocarcinoma (5; 4.76%)<br>Tubular adenoma (4; 3.81%)<br>Cholangiocarcinoma (4; 3.81%)<br>Leiomyosarcoma (4; 3.81%)<br>Salivary gland carcinoma (3; 2.86%)<br>Tubulopapillary adenocarcinoma (2; 1.90%)<br>Papillary adenocarcinoma (2; 1.90%)<br>Hepatic carcinoid (1; 0.95%)<br>Extraskeletal osteosarcoma (1; 0.95%)<br>Signet-ring cell carcinoma (1; 0.95%)<br>Hepatocellular adenoma (1; 0.95%)<br>Hepatic lymphoma (1; 0.95%)<br>Plasmacytoma (1; 0.95%)<br>Acinar adenocarcinoma (1; 0.95%)<br>Biliary adenoma (1; 0.95%)<br>Papillary adenoma (1; 0.95%)
Musculoskeletal system	40 (0.63%)	Osteosarcoma (34; 85.00%)<br>Chondrosarcoma (6; 15.00%)
Urinary system	25 (0.39%)	Urothelial cell carcinoma (12; 48.00%)<br>Renal adenocarcinoma (5; 20.00%)<br>Renal haemangioma (1; 4.00%)<br>Vesical leiomyoma (1; 4.00%)<br>Renal lymphoma (1; 4.00%)

		Nephroblastoma (1; 4.00%)<br>Urothelial papilloma (1; 4.00%)<br>Renal adenoma (1; 4.00%)<br>Haemangiosarcoma (1; 4.00%)<br>Renal fibrosarcoma (1; 4.00%)
Neuroendocrine	21 (0.33%)	Thyroid carcinoma (13; 61.90%)<br>Chemodectoma (3; 14.29%)<br>Pheochromocytoma (2; 9.52%)<br>Adrenocortical carcinoma (2; 9.52%)<br>Insulinoma (1; 4.76%)
Respiratory system	6 (0.09%)	Pulmonary adenocarcinoma (3; 50.00%)<br>Nasal adenocarcinoma (2; 33.33%)<br>Nasal transitional cell carcinoma (1; 16.67%)
Body cavities	1 (0.02%)	Haemangiosarcoma (1; 100.00%)
