# Supplementary material for: Every Tumour Counts: A Comprehensive Overview of Canine Oncology in Portugal
Source: Animals (Basel). 2025 Dec 23;16(1):35. doi: 10.3390/ani16010035 (PMC12784951; doi:10.3390/ani16010035)
Supplement: Supplementary file 1 [file animals-16-00035-s001.zip › Table S2.pdf]

Supplementary Table S2. Distribution of canine tumours by breed, anatomical location, and diagnosis: totals (*n*; % of all cases) and within-location diagnosis (*n*; %).

Hierarchical category (breed–anatomical location–diagnosis)	Cases (*n*)	Within-breed (%)
**Afghan Hound**	**2**	**0.03%**
**Cutaneous and soft tissue**	**1**	**50.00%**
Lipoma	1	100.00%
**Male reproductive system**	**1**	**50.00%**
Seminoma	1	100.00%
**Airedale Terrier**	**2**	**0.03%**
**Cutaneous and soft tissue**	**1**	**50.00%**
Schwannoma	1	100.00%
**Mammary**	**1**	**50.00%**
Malignant mammary tumour	1	100.00%
**Akita**	**6**	**0.09%**
**Cutaneous and soft tissue**	**5**	**83.33%**
Hepatoid epithelioma	2	40.00%
Hemangiosarcoma	1	20.00%
Hepatoid adenoma	1	20.00%
Squamous papilloma	1	20.00%
**Mammary**	**1**	**16.67%**
Malignant mammary tumour	1	100.00%
**Alaskan Malamute**	**2**	**0.03%**
**Female reproductive system**	**1**	**50.00%**
Leiomyoma	1	100.00%
**Gastrointestinal tract**	**1**	**50.00%**
Leiomyosarcoma	1	100.00%
**Alentejo Mastiff**	**33**	**0.52%**
**Cutaneous and soft tissue**	**27**	**81.82%**
Trichoblastoma	4	14.81%
Lipoma	4	14.81%
Apocrine adenoma	2	7.41%
Hepatoid adenoma	2	7.41%
Trichoepithelioma	2	7.41%
Anal sac adenocarcinoma	1	3.70%
Apocrine adenocarcinoma	1	3.70%
Apocrine ductal adenocarcinoma	1	3.70%
Fibrosarcoma	1	3.70%
Hemangiosarcoma	1	3.70%
Hemangiosarcoma	1	3.70%
Hepatoid epithelioma	1	3.70%
Infundibular keratinizing acanthoma	1	3.70%
Mast cell tumor (Grade II)	1	3.70%
Mast cell tumor (Grade III)	1	3.70%
Melanoma	1	3.70%
Schwannoma	1	3.70%
Squamous papilloma	1	3.70%
**Gastrointestinal tract**	**1**	**3.03%**

Intestinal lymphoma	1	100.00%
**Haemolymphatic system**	**1**	**3.03%**
Splenic hemangiosarcoma	1	100.00%
**Male reproductive system**	**1**	**3.03%**
Mixed germ cell sex-cord stromal tumor	1	100.00%
**Mammary**	**3**	**9.09%**
Malignant mammary tumour	3	100.00%
**American Bully**	**2**	**0.03%**
**Cutaneous and soft tissue**	**1**	**50.00%**
Fibroma	1	100.00%
**Musculoskeletal system**	**1**	**50.00%**
Osteosarcoma	1	100.00%
**American Pitbull**	**70**	**1.10%**
**Body cavity**	**1**	**1.43%**
Heart Hemangiosarcoma	1	100.00%
**Cutaneous and soft tissue**	**59**	**84.29%**
Mast cell tumor (Grade II)	9	15.25%
Squamous cell carcinoma	6	10.17%
Hemangiosarcoma	5	8.47%
Hepatoid adenoma	4	6.78%
Histiocytoma	4	6.78%
Melanoma	4	6.78%
Mast cell tumor (Grade I)	3	5.08%
Mast cell tumor (Grade III)	3	5.08%
Melanocytoma	3	5.08%
Perivascular wall tumor	3	5.08%
Hemangioma	2	3.39%
Hepatoid epithelioma	2	3.39%
Squamous papilloma	2	3.39%
Apocrine adenoma	1	1.69%
Fibroma	1	1.69%
Fibrosarcoma	1	1.69%
Lymphoma	1	1.69%
Malignant plasmacytoma	1	1.69%
Matrical carcinoma	1	1.69%
Sebaceous adenoma	1	1.69%
Subcutaneous mast cell tumor	1	1.69%
Trichoepithelioma	1	1.69%
**Mammary**	**6**	**8.57%**
Malignant mammary tumour	4	66.67%
Benign mammary tumour	2	33.33%
**Neuroendocrine**	**1**	**1.43%**
Thyroid carcinoma	1	100.00%
**Ocular system**	**1**	**1.43%**
Meibomian adenoma	1	100.00%
**Oral cavity**	**2**	**2.86%**
Melanoma	1	50.00%
Peripheral odontogenic fibroma	1	50.00%

**American Staffordshire Terrier**	**19**	**0.30%**
**Cutaneous and soft tissue**	**16**	**84.21%**
Mast cell tumor (Grade II)	8	50.00%
Hemangiosarcoma	2	12.50%
Hemangioma	1	6.25%
Histiocytoma	1	6.25%
Mast cell tumor (Grade I)	1	6.25%
Mast cell tumor (Grade III)	1	6.25%
Squamous cell carcinoma	1	6.25%
Trichoepithelioma	1	6.25%
**Female reproductive system**	**1**	**5.26%**
Dysgerminoma	1	100.00%
**Mammary**	**2**	**10.53%**
Malignant mammary tumour	2	100.00%
**Australian Shepherd**	**6**	**0.09%**
**Cutaneous and soft tissue**	**4**	**66.67%**
Perivascular wall tumor	2	50.00%
Apocrine adenocarcinoma	1	25.00%
Mast cell tumor (Grade III)	1	25.00%
**Female reproductive system**	**2**	**33.33%**
Leiomyoma	1	50.00%
Leiomyosarcoma	1	50.00%
**Basset fauve de Bretagne**	**1**	**0.02%**
**Cutaneous and soft tissue**	**1**	**100.00%**
Mast cell tumor (Grade II)	1	100.00%
**Basset Hound**	**34**	**0.53%**
**Cutaneous and soft tissue**	**29**	**85.29%**
Trichoepithelioma	15	51.72%
Pilomatricoma	5	17.24%
Hemangiosarcoma	2	6.90%
Malignant trichoepithelioma	2	6.90%
Mast cell tumor (Grade I)	2	6.90%
Hemangioma	1	3.45%
Mast cell tumor (Grade II)	1	3.45%
Matrical carcinoma	1	3.45%
**Mammary**	**1**	**2.94%**
Malignant mammary tumour	1	100.00%
**Neuroendocrine**	**1**	**2.94%**
Thyroid carcinoma	1	100.00%
**Ocular system**	**2**	**5.88%**
Meibomian adenoma	1	50.00%
Meibomian epithelioma	1	50.00%
**Oral cavity**	**1**	**2.94%**
Peripheral odontogenic fibroma	1	100.00%
**Beagle**	**136**	**2.14%**
**Cutaneous and soft tissue**	**100**	**73.53%**
Histiocytoma	12	12.00%
Lipoma	12	12.00%

Hepatoid adenoma	10	10.00%
Perivascular wall tumor	9	9.00%
Mast cell tumor (Grade II)	8	8.00%
Squamous papilloma	6	6.00%
Hemangiosarcoma	5	5.00%
Subcutaneous mast cell tumor	5	5.00%
Hemangioma	3	3.00%
Infundibular keratinizing acanthoma	3	3.00%
Mast cell tumor (Grade III)	3	3.00%
Plasmacytoma	3	3.00%
Trichoblastoma	3	3.00%
Fibrosarcoma	2	2.00%
Mast cell tumor (Grade I)	2	2.00%
Pilomatricoma	2	2.00%
Sebaceous epithelioma	2	2.00%
Squamous cell carcinoma	2	2.00%
Trichoepithelioma	2	2.00%
Anal sac adenocarcinoma	1	1.00%
Apocrine ductal adenocarcinoma	1	1.00%
Fibroma	1	1.00%
Hepatoid epithelioma	1	1.00%
Melanocytoma	1	1.00%
Sebaceous adenoma	1	1.00%
**Gastrointestinal tract**	**4**	**2.94%**
GIST	1	25.00%
Hepatocelular carcinoma	1	25.00%
Intestinal adenocarcinoma	1	25.00%
Intestinal adenoma	1	25.00%
**Haemolymphatic system**	**3**	**2.21%**
Lymphoma	2	66.67%
Splenic lymphoma	1	33.33%
**Male reproductive system**	**3**	**2.21%**
Mixed germ cell sex-cord stromal tumor	2	66.67%
Intersticial cell tumor	1	33.33%
**Mammary**	**20**	**14.71%**
Malignant mammary tumour	17	85.00%
Benign mammary tumour	3	15.00%
**Ocular system**	**4**	**2.94%**
Meibomian adenoma	4	100.00%
**Oral cavity**	**1**	**0.74%**
Melanoma	1	100.00%
**Urinary system**	**1**	**0.74%**
Hemangiosarcoma	1	100.00%
**Belgian Malinois**	**3**	**0.05%**
**Cutaneous and soft tissue**	**1**	**33.33%**
Pilomatricoma	1	100.00%
**Mammary**	**2**	**66.67%**
Benign mammary tumour	1	50.00%

Malignant mammary tumour	1	50.00%
**Belgian Shepherd**	**26**	**0.41%**
**Cutaneous and soft tissue**	**16**	**61.54%**
Hepatoid adenoma	3	18.75%
Hepatoid epithelioma	2	12.50%
Mast cell tumor (Grade II)	2	12.50%
Hemangioma	1	6.25%
Hemangiosarcoma	1	6.25%
Histiocytoma	1	6.25%
Mast cell tumor (Grade I)	1	6.25%
Myxosarcoma	1	6.25%
Perivascular wall tumor	1	6.25%
Squamous papilloma	1	6.25%
Trichoblastoma	1	6.25%
Trichoepithelioma	1	6.25%
**Mammary**	**8**	**30.77%**
Malignant mammary tumour	5	62.50%
Benign mammary tumour	3	37.50%
**Musculoskeletal system**	**1**	**3.85%**
Osteosarcoma	1	100.00%
**Oral cavity**	**1**	**3.85%**
Melanoma	1	100.00%
**Bichon Frise**	**10**	**0.16%**
**Cutaneous and soft tissue**	**4**	**40.00%**
Fibrosarcoma	1	25.00%
Hepatoid epithelioma	1	25.00%
Subcutaneous mast cell tumor	1	25.00%
Trichoblastoma	1	25.00%
**Gastrointestinal tract**	**1**	**10.00%**
Hepatic carcinoid	1	100.00%
**Mammary**	**5**	**50.00%**
Malignant mammary tumour	4	80.00%
Benign mammary tumour	1	20.00%
**Bobtail**	**8**	**0.13%**
**Cutaneous and soft tissue**	**5**	**62.50%**
Sebaceous adenoma	2	40.00%
Apocrine ductal adenocarcinoma	1	20.00%
Infundibular keratinizing acanthoma	1	20.00%
Trichoepithelioma	1	20.00%
**Neuroendocrine**	**1**	**12.50%**
Thyroid carcinoma	1	100.00%
**Ocular system**	**1**	**12.50%**
Meibomian adenoma	1	100.00%
**Oral cavity**	**1**	**12.50%**
Peripheral odontogenic fibroma	1	100.00%
**Boerboel**	**2**	**0.03%**
**Cutaneous and soft tissue**	**2**	**100.00%**
Hemangioma	1	50.00%

Mast cell tumor (Grade III)	1	50.00%
**Bordeaux Mastiff**	**7**	**0.11%**
**Cutaneous and soft tissue**	**3**	**42.86%**
Trichoepithelioma	2	66.67%
Hemangiosarcoma	1	33.33%
**Female reproductive system**	**1**	**14.29%**
Ovarian carcinoma	1	100.00%
**Haemolymphatic system**	**1**	**14.29%**
Lymphoma	1	100.00%
**Oral cavity**	**2**	**28.57%**
Peripheral odontogenic fibroma	2	100.00%
**Border Collie**	**17**	**0.27%**
**Cutaneous and soft tissue**	**10**	**58.82%**
Lipoma	3	30.00%
Fibroma	1	10.00%
Hemangiosarcoma	1	10.00%
Hepatoid adenoma	1	10.00%
Hepatoid epithelioma	1	10.00%
Myxosarcoma	1	10.00%
Perivascular wall tumor	1	10.00%
Pilomatricoma	1	10.00%
**Female reproductive system**	**1**	**5.88%**
Ovarian carcinoma	1	100.00%
**Male reproductive system**	**3**	**17.65%**
Intersticial cell tumor	1	33.33%
Seminoma	1	33.33%
Sertoli cell tumor	1	33.33%
**Mammary**	**2**	**11.76%**
Malignant mammary tumour	2	100.00%
**Ocular system**	**1**	**5.88%**
Meibomian adenoma	1	100.00%
**Boston terrier**	**3**	**0.05%**
**Cutaneous and soft tissue**	**2**	**66.67%**
Mast cell tumor (Grade II)	1	50.00%
Squamous cell carcinoma	1	50.00%
**Female reproductive system**	**1**	**33.33%**
Leiomyoma	1	100.00%
**Bouvier Bernois**	**10**	**0.16%**
**Cutaneous and soft tissue**	**6**	**60.00%**
Mast cell tumor (Grade II)	2	33.33%
Lipoma	1	16.67%
Melanoma	1	16.67%
Schwannoma	1	16.67%
Trichoblastoma	1	16.67%
**Male reproductive system**	**1**	**10.00%**
Seminoma	1	100.00%
**Mammary**	**1**	**10.00%**
Malignant mammary tumour	1	100.00%

**Oral cavity**	**2**	**20.00%**
Peripheral odontogenic fibroma	2	100.00%
**Bouvier des Flandres**	**1**	**0.02%**
**Cutaneous and soft tissue**	**1**	**100.00%**
Squamous cell carcinoma	1	100.00%
**Boxer**	**181**	**2.85%**
**Cutaneous and soft tissue**	**137**	**75.69%**
Mast cell tumor (Grade II)	40	29.20%
Histiocytoma	16	11.68%
Perivascular wall tumor	12	8.76%
Hemangiosarcoma	10	7.30%
Mast cell tumor (Grade I)	9	6.57%
Mast cell tumor (Grade III)	7	5.11%
Squamous cell carcinoma	5	3.65%
Subcutaneous mast cell tumor	5	3.65%
Lipoma	4	2.92%
Melanocytoma	4	2.92%
Fibrosarcoma	3	2.19%
Melanoma	3	2.19%
Fibroma	2	1.46%
Hemangioma	2	1.46%
Hemangiosarcoma	2	1.46%
Plasmacytoma	2	1.46%
Schwannoma	2	1.46%
Basal cell carcinoma	1	0.73%
Histiocytic sarcoma	1	0.73%
Inverted squamous papilloma	1	0.73%
Mesothelioma	1	0.73%
Myxoma	1	0.73%
Pilomatricoma	1	0.73%
Sebaceous adenoma	1	0.73%
Sebaceous epithelioma	1	0.73%
Trichoepithelioma	1	0.73%
**Gastrointestinal tract**	**3**	**1.66%**
Intestinal adenocarcinoma	2	66.67%
Intestinal adenoma	1	33.33%
**Haemolymphatic system**	**6**	**3.31%**
Splenic hemangiosarcoma	4	66.67%
Splenic lymphoma	1	16.67%
Lymphoma	1	16.67%
**Male reproductive system**	**7**	**3.87%**
Intersticial cell tumor	3	42.86%
Seminoma	3	42.86%
Mixed germ cell sex-cord stromal tumor	1	14.29%
**Mammary**	**14**	**7.73%**
Malignant mammary tumour	11	78.57%
Benign mammary tumour	3	21.43%
**Musculoskeletal system**	**1**	**0.55%**

Osteosarcoma	1	100.00%
**Neuroendocrine**	**2**	**1.10%**
Chemodectoma	1	50.00%
Thyroid carcinoma	1	50.00%
**Ocular system**	**1**	**0.55%**
Meibomian adenoma	1	100.00%
**Oral cavity**	**10**	**5.52%**
Peripheral odontogenic fibroma	9	90.00%
Ameloblastic carcinoma	1	10.00%
**Brazilian Mastiff**	**1**	**0.02%**
**Mammary**	**1**	**100.00%**
Malignant mammary tumour	1	100.00%
**Briard**	**2**	**0.03%**
**Cutaneous and soft tissue**	**2**	**100.00%**
Pilomatricoma	1	50.00%
Squamous cell carcinoma	1	50.00%
**Bull Mastiff**	**5**	**0.08%**
**Cutaneous and soft tissue**	**3**	**60.00%**
Basosquamous carcinoma	1	33.33%
Mast cell tumor (Grade III)	1	33.33%
Trichoepithelioma	1	33.33%
**Male reproductive system**	**1**	**20.00%**
Seminoma	1	100.00%
**Musculoskeletal system**	**1**	**20.00%**
Osteosarcoma	1	100.00%
**Bull Terrier**	**27**	**0.42%**
**Cutaneous and soft tissue**	**19**	**70.37%**
Squamous cell carcinoma	9	47.37%
Histiocytoma	3	15.79%
Lipoma	3	15.79%
Mast cell tumor (Grade I)	1	5.26%
Mast cell tumor (Grade III)	1	5.26%
Melanoma	1	5.26%
Subcutaneous mast cell tumor	1	5.26%
**Mammary**	**5**	**18.52%**
Malignant mammary tumour	5	100.00%
**Ocular system**	**1**	**3.70%**
Meibomian adenoma	1	100.00%
**Oral cavity**	**2**	**7.41%**
Peripheral odontogenic fibroma	2	100.00%
**Cairn Terrier**	**10**	**0.16%**
**Cutaneous and soft tissue**	**7**	**70.00%**
Plasmacytoma	2	28.57%
Ceruminous adenoma	1	14.29%
Cutaneous histiocytosis	1	14.29%
Infundibular keratinizing acanthoma	1	14.29%
Perivascular wall tumor	1	14.29%
Squamous cell carcinoma	1	14.29%

**Gastrointestinal tract**	**1**	**10.00%**
Intestinal adenoma	1	100.00%
**Male reproductive system**	**1**	**10.00%**
Intersticial cell tumor	1	100.00%
**Oral cavity**	**1**	**10.00%**
Peripheral odontogenic fibroma	1	100.00%
**Cane Corso**	**17**	**0.27%**
**Cutaneous and soft tissue**	**13**	**76.47%**
Histiocytoma	3	23.08%
Mast cell tumor (Grade III)	3	23.08%
Mast cell tumor (Grade II)	2	15.38%
Trichoepithelioma	2	15.38%
Hemangiosarcoma	1	7.69%
Melanocytoma	1	7.69%
Plasmacytoma	1	7.69%
**Mammary**	**3**	**17.65%**
Malignant mammary tumour	3	100.00%
**Oral cavity**	**1**	**5.88%**
Peripheral odontogenic fibroma	1	100.00%
**Castro Laboreiro Dog**	**9**	**0.14%**
**Cutaneous and soft tissue**	**5**	**55.56%**
Apocrine adenoma	1	20.00%
Apocrine ductal adenocarcinoma	1	20.00%
Lipoma	1	20.00%
Mast cell tumor (Grade II)	1	20.00%
Melanoma	1	20.00%
**Male reproductive system**	**1**	**11.11%**
Intersticial cell tumor	1	100.00%
**Mammary**	**3**	**33.33%**
Malignant mammary tumour	3	100.00%
**Cavalier King Charles**	**7**	**0.11%**
**Cutaneous and soft tissue**	**3**	**42.86%**
Trichoblastoma	2	66.67%
Neuroblastoma	1	33.33%
**Male reproductive system**	**1**	**14.29%**
Mixed germ cell sex-cord stromal tumor	1	100.00%
**Mammary**	**1**	**14.29%**
Malignant mammary tumour	1	100.00%
**Ocular system**	**1**	**14.29%**
Meibomian epithelioma	1	100.00%
**Oral cavity**	**1**	**14.29%**
Melanoma	1	100.00%
**Chihuahua**	**35**	**0.55%**
**Cutaneous and soft tissue**	**15**	**42.86%**
Hepatoid adenoma	2	13.33%
Histiocytoma	2	13.33%
Squamous cell carcinoma	2	13.33%
Ganglioneuroma	1	6.67%

Hemangioma	1	6.67%
Hemangiosarcoma	1	6.67%
Infundibular keratinizing acanthoma	1	6.67%
Mast cell tumor (Grade II)	1	6.67%
Melanoma	1	6.67%
Perivascular wall tumor	1	6.67%
Trichoblastoma	1	6.67%
Trichoepithelioma	1	6.67%
**Female reproductive system**	**1**	**2.86%**
Leiomyoma	1	100.00%
**Gastrointestinal tract**	**1**	**2.86%**
Leiomyosarcoma	1	100.00%
**Haemolymphatic system**	**1**	**2.86%**
Splenic hemangiosarcoma	1	100.00%
**Male reproductive system**	**1**	**2.86%**
Seminoma	1	100.00%
**Mammary**	**16**	**45.71%**
Malignant mammary tumour	12	75.00%
Benign mammary tumour	4	25.00%
**Chinese Crested Dog**	**1**	**0.02%**
**Cutaneous and soft tissue**	**1**	**100.00%**
Plasmacytoma	1	100.00%
**Chow-Chow**	**11**	**0.17%**
**Cutaneous and soft tissue**	**6**	**54.55%**
Anal sac adenocarcinoma	1	16.67%
Apocrine adenocarcinoma	1	16.67%
Hepatoid adenoma	1	16.67%
Melanocytoma	1	16.67%
Perivascular wall tumor	1	16.67%
Squamous cell carcinoma	1	16.67%
**Female reproductive system**	**1**	**9.09%**
Benign ovarian tumor	1	100.00%
**Mammary**	**3**	**27.27%**
Malignant mammary tumour	3	100.00%
**Ocular system**	**1**	**9.09%**
Meibomian adenoma	1	100.00%
**Cocker Spaniel**	**152**	**2.39%**
**Cutaneous and soft tissue**	**83**	**54.61%**
Sebaceous epithelioma	11	13.25%
Lipoma	8	9.64%
Sebaceous adenoma	8	9.64%
Histiocytoma	7	8.43%
Melanoma	7	8.43%
Fibrosarcoma	4	4.82%
Perivascular wall tumor	4	4.82%
Hemangioma	3	3.61%
Hemangiosarcoma	3	3.61%
Melanocytoma	3	3.61%

Plasmacytoma	3	3.61%
Trichoblastoma	3	3.61%
Hepatoid adenoma	2	2.41%
Infundibular keratinizing acanthoma	2	2.41%
Squamous papilloma	2	2.41%
Subcutaneous mast cell tumor	2	2.41%
Trichoepithelioma	2	2.41%
Anal sac adenocarcinoma	1	1.20%
Apocrine adenoma	1	1.20%
Ceruminous adenocarcinoma	1	1.20%
Ceruminous adenoma	1	1.20%
Malignant plasmacytoma	1	1.20%
Mast cell tumor (Grade I)	1	1.20%
Mast cell tumor (Grade II)	1	1.20%
Mast cell tumor (Grade III)	1	1.20%
Squamous cell carcinoma	1	1.20%
**Female reproductive system**	**2**	**1.32%**
Benign ovarian tumor	1	50.00%
Leiomyoma	1	50.00%
**Gastrointestinal tract**	**4**	**2.63%**
Intestinal adenocarcinoma	3	75.00%
Hepatocelular carcinoma	1	25.00%
**Haemolymphatic system**	**3**	**1.97%**
Lymphoma	1	33.33%
Splenic lymphoma	1	33.33%
Splenic Myelolipoma	1	33.33%
**Male reproductive system**	**5**	**3.29%**
Sertoli cell tumor	2	40.00%
Intersticial cell tumor	1	20.00%
Seminoma	1	20.00%
Seminoma	1	20.00%
**Mammary**	**49**	**32.24%**
Malignant mammary tumour	37	75.51%
Benign mammary tumour	12	24.49%
**Ocular system**	**1**	**0.66%**
Meibomian adenoma	1	100.00%
**Oral cavity**	**4**	**2.63%**
Melanoma	2	50.00%
Peripheral odontogenic fibroma	1	25.00%
Plasmacytoma	1	25.00%
**Urinary system**	**1**	**0.66%**
Urothelial cell carcinoma	1	100.00%
**Czech Wolfdog**	**1**	**0.02%**
**Gastrointestinal tract**	**1**	**100.00%**
Intestinal lymphoma	1	100.00%
**Dachshund**	**3**	**0.05%**
**Cutaneous and soft tissue**	**2**	**66.67%**
Mast cell tumor (Grade I)	1	50.00%

Infundibular keratinizing acanthoma	1	50.00%
**Oral cavity**	**1**	**33.33%**
Peripheral odontogenic fibroma	1	100.00%
**Dalmatian**	**20**	**0.31%**
**Cutaneous and soft tissue**	**13**	**65.00%**
Lipoma	5	38.46%
Hemangiosarcoma	2	15.38%
Squamous cell carcinoma	2	15.38%
Fibroma	1	7.69%
Histiocytoma	1	7.69%
Squamous papilloma	1	7.69%
Trichoepithelioma	1	7.69%
**Haemolymphatic system**	**1**	**5.00%**
Lymphoma	1	100.00%
**Male reproductive system**	**1**	**5.00%**
Intersticial cell tumor	1	100.00%
**Mammary**	**4**	**20.00%**
Malignant mammary tumour	4	100.00%
**Oral cavity**	**1**	**5.00%**
Peripheral odontogenic fibroma	1	100.00%
**Doberman**	**25**	**0.39%**
**Cutaneous and soft tissue**	**16**	**64.00%**
Histiocytoma	4	25.00%
Perivascular wall tumor	3	18.75%
Plasmacytoma	2	12.50%
Fibrosarcoma	1	6.25%
Hemangioma	1	6.25%
Mast cell tumor (Grade I)	1	6.25%
Mast cell tumor (Grade II)	1	6.25%
Mast cell tumor (Grade III)	1	6.25%
Melanocytoma	1	6.25%
Squamous papilloma	1	6.25%
**Gastrointestinal tract**	**1**	**4.00%**
Intestinal adenoma	1	100.00%
**Haemolymphatic system**	**2**	**8.00%**
Lymphoma	2	100.00%
**Mammary**	**4**	**16.00%**
Malignant mammary tumour	3	75.00%
Benign mammary tumour	1	25.00%
**Musculoskeletal system**	**1**	**4.00%**
Chondrosarcoma	1	100.00%
**Ocular system**	**1**	**4.00%**
Meibomian adenoma	1	100.00%
**Dogo Argentino**	**7**	**0.11%**
**Cutaneous and soft tissue**	**5**	**71.43%**
Hemangiosarcoma	2	40.00%
Hemangioma	1	20.00%
Squamous cell carcinoma	1	20.00%

Subcutaneous mast cell tumor	1	20.00%
**Male reproductive system**	**1**	**14.29%**
Interstitial cell tumor	1	100.00%
**Mammary**	**1**	**14.29%**
Malignant mammary tumour	1	100.00%
**Dogo Canario**	**1**	**0.02%**
**Cutaneous and soft tissue**	**1**	**100.00%**
Anal sac adenocarcinoma	1	100.00%
**Drathaar**	**1**	**0.02%**
**Cutaneous and soft tissue**	**1**	**100.00%**
Perivascular wall tumor	1	100.00%
**English Bulldog**	**16**	**0.25%**
**Cutaneous and soft tissue**	**8**	**50.00%**
Squamous papilloma	2	25.00%
Hemangioma	1	12.50%
Histiocytoma	1	12.50%
Infundibular keratinizing acanthoma	1	12.50%
Mast cell tumor (Grade II)	1	12.50%
Sebaceous adenoma	1	12.50%
Squamous cell carcinoma	1	12.50%
**Female reproductive system**	**2**	**12.50%**
Benign ovarian tumor	2	100.00%
**Gastrointestinal tract**	**2**	**12.50%**
Cholangiocarcinoma	1	50.00%
Intestinal adenoma	1	50.00%
**Male reproductive system**	**1**	**6.25%**
Seminoma	1	100.00%
**Mammary**	**1**	**6.25%**
Malignant mammary tumour	1	100.00%
**Ocular system**	**1**	**6.25%**
Meibomian adenoma	1	100.00%
**Oral cavity**	**1**	**6.25%**
Peripheral odontogenic fibroma	1	100.00%
**English Setter**	**29**	**0.46%**
**Cutaneous and soft tissue**	**21**	**72.41%**
Mast cell tumor (Grade II)	8	38.10%
Histiocytoma	2	9.52%
Infundibular keratinizing acanthoma	2	9.52%
Mast cell tumor (Grade I)	2	9.52%
Subcutaneous mast cell tumor	2	9.52%
Hemangioma	1	4.76%
Hepatoid adenoma	1	4.76%
Lipoma	1	4.76%
Trichoblastoma	1	4.76%
Trichoepithelioma	1	4.76%
**Male reproductive system**	**1**	**3.45%**
Interstitial cell tumor	1	100.00%
**Mammary**	**7**	**24.14%**

Malignant mammary tumour	6	85.71%
Benign mammary tumour	1	14.29%
**English Springer Spaniel**	**3**	**0.05%**
**Cutaneous and soft tissue**	**3**	**100.00%**
Mast cell tumor (Grade II)	1	33.33%
Pilomatricoma	1	33.33%
Sebaceous adenoma	1	33.33%
**Epagneul Breton**	**28**	**0.44%**
**Cutaneous and soft tissue**	**12**	**42.86%**
Fibrosarcoma	2	16.67%
Lipoma	2	16.67%
Mast cell tumor (Grade II)	2	16.67%
Fibroma	1	8.33%
Hemangioma	1	8.33%
Hemangiosarcoma	1	8.33%
Hepatoid adenoma	1	8.33%
Mast cell tumor (Grade III)	1	8.33%
Melanocytoma	1	8.33%
**Male reproductive system**	**1**	**3.57%**
Interstицial cell tumor	1	100.00%
**Mammary**	**12**	**42.86%**
Malignant mammary tumour	9	75.00%
Benign mammary tumour	3	25.00%
**Ocular system**	**2**	**7.14%**
Meibomian adenoma	2	100.00%
**Urinary system**	**1**	**3.57%**
Renal adenocarcinoma	1	100.00%
**Estrela Mountain Dog**	**49**	**0.77%**
**Cutaneous and soft tissue**	**34**	**69.39%**
Perivascular wall tumor	5	14.71%
Apocrine adenocarcinoma	4	11.76%
Trichoepithelioma	4	11.76%
Infundibular keratinizing acanthoma	3	8.82%
Apocrine adenoma	2	5.88%
Hepatoid adenoma	2	5.88%
Trichoblastoma	2	5.88%
Fibrosarcoma	1	2.94%
Hemangioma	1	2.94%
Hepatoid epithelioma	1	2.94%
Histiocytoma	1	2.94%
Lipoma	1	2.94%
Mast cell tumor (Grade II)	1	2.94%
Matrical carcinoma	1	2.94%
Melanoma	1	2.94%
Myxosarcoma	1	2.94%
Pilomatricoma	1	2.94%
Plasmacytoma	1	2.94%
Squamous cell carcinoma	1	2.94%

**Female reproductive system**	**4**	**8.16%**
Benign ovarian tumor	4	100.00%
**Male reproductive system**	**5**	**10.20%**
Intersticial cell tumor	2	40.00%
Sertoli cell tumor	2	40.00%
Mixed germ cell sex-cord stromal tumor	1	20.00%
**Mammary**	**4**	**8.16%**
Malignant mammary tumour	4	100.00%
**Neuroendocrine**	**1**	**2.04%**
Pheochromocytoma	1	100.00%
**Oral cavity**	**1**	**2.04%**
Peripheral odontogenic fibroma	1	100.00%
**Flat Coated Retriever**	**1**	**0.02%**
**Cutaneous and soft tissue**	**1**	**100.00%**
Histiocytoma	1	100.00%
**Fox Terrier**	**6**	**0.09%**
**Cutaneous and soft tissue**	**3**	**50.00%**
Hemangioma	1	33.33%
Sebaceous adenoma	1	33.33%
Squamous cell carcinoma	1	33.33%
**Male reproductive system**	**1**	**16.67%**
Seminoma	1	100.00%
**Mammary**	**1**	**16.67%**
Malignant mammary tumour	1	100.00%
**Urinary system**	**1**	**16.67%**
Urothelial cell carcinoma	1	100.00%
**French Bulldog**	**295**	**4.64%**
**Cutaneous and soft tissue**	**215**	**72.88%**
Histiocytoma	71	33.02%
Mast cell tumor (Grade II)	47	21.86%
Mast cell tumor (Grade III)	25	11.63%
Mast cell tumor (Grade I)	12	5.58%
Squamous papilloma	7	3.26%
Squamous cell carcinoma	5	2.33%
Subcutaneous mast cell tumor	5	2.33%
Hemangioma	4	1.86%
Hemangiosarcoma	4	1.86%
Perivascular wall tumor	4	1.86%
Plasmacytoma	4	1.86%
Fibroma	3	1.40%
Fibrosarcoma	3	1.40%
Melanoma	3	1.40%
Hemangiosarcoma	2	0.93%
Hepatoid epithelioma	2	0.93%
Myxosarcoma	2	0.93%
Pilomatricoma	2	0.93%
Trichoepithelioma	2	0.93%
Apocrine adenoma	1	0.47%

Apocrine ductal adenocarcinoma	1	0.47%
Cutaneous histiocytosis	1	0.47%
Infundibular keratinizing acanthoma	1	0.47%
Lipoma	1	0.47%
Lymphoma	1	0.47%
Malignant plasmacytoma	1	0.47%
Mesothelioma	1	0.47%
**Female reproductive system**	**7**	**2.37%**
Benign ovarian tumor	5	71.43%
Ovarian carcinoma	1	14.29%
Leiomyoma	1	14.29%
**Gastrointestinal tract**	**4**	**1.36%**
Intestinal adenoma	3	75.00%
Intestinal lymphoma	1	25.00%
**Haemolymphatic system**	**7**	**2.37%**
Splenic hemangiosarcoma	6	85.71%
Lymphoma	1	14.29%
**Male reproductive system**	**7**	**2.37%**
Seminoma	3	42.86%
Sertoli cell tumor	3	42.86%
Seminoma	1	14.29%
**Mammary**	**36**	**12.20%**
Malignant mammary tumour	28	77.78%
Benign mammary tumour	8	22.22%
**Musculoskeletal system**	**1**	**0.34%**
Chondrosarcoma	1	100.00%
**Neuroendocrine**	**1**	**0.34%**
Adrenocortical carcinoma	1	100.00%
**Ocular system**	**5**	**1.69%**
Meibomian adenoma	4	80.00%
Meibomian epithelioma	1	20.00%
**Oral cavity**	**12**	**4.07%**
Peripheral odontogenic fibroma	9	75.00%
Acanthomatous ameloblastoma	3	25.00%
**German Shepherd**	**249**	**3.92%**
**Cutaneous and soft tissue**	**129**	**51.81%**
Infundibular keratinizing acanthoma	21	16.28%
Perivascular wall tumor	14	10.85%
Hemangioma	13	10.08%
Trichoblastoma	11	8.53%
Trichoepithelioma	8	6.20%
Hepatoid adenoma	7	5.43%
Hemangiosarcoma	5	3.88%
Melanocytoma	5	3.88%
Lipoma	4	3.10%
Apocrine adenocarcinoma	3	2.33%
Apocrine adenoma	3	2.33%
Mast cell tumor (Grade II)	3	2.33%

Melanoma	3	2.33%
Basal cell carcinoma	2	1.55%
Fibrosarcoma	2	1.55%
Hemangiosarcoma	2	1.55%
Hepatoid epithelioma	2	1.55%
Histiocytoma	2	1.55%
Myxosarcoma	2	1.55%
Pilomatricoma	2	1.55%
Squamous papilloma	2	1.55%
Basosquamous carcinoma	1	0.78%
Ceruminous adenocarcinoma	1	0.78%
Ductal adenocarcinoma	1	0.78%
Extraskeletal chondrosarcoma	1	0.78%
Extraskeletal osteosarcoma	1	0.78%
Inverted squamous papilloma	1	0.78%
Liposarcoma	1	0.78%
Malignant trichoepithelioma	1	0.78%
Mast cell tumor (Grade I)	1	0.78%
Mast cell tumor (Grade III)	1	0.78%
Squamous cell carcinoma	1	0.78%
Subcutaneous mast cell tumor	1	0.78%
Tricholemmoma	1	0.78%
**Female reproductive system**	**1**	**0.40%**
Leiomyoma	1	100.00%
**Gastrointestinal tract**	**3**	**1.20%**
Cholangiocarcinoma	1	33.33%
Intestinal adenocarcinoma	1	33.33%
Intestinal adenoma	1	33.33%
**Haemolymphatic system**	**11**	**4.42%**
Splenic hemangiosarcoma	11	100.00%
**Male reproductive system**	**11**	**4.42%**
Seminoma	5	45.45%
Mixed germ cell sex-cord stromal tumor	3	27.27%
Intersticial cell tumor	1	9.09%
Seminoma	1	9.09%
Sertoli cell tumor	1	9.09%
**Mammary**	**84**	**33.73%**
Malignant mammary tumour	73	86.90%
Benign mammary tumour	11	13.10%
**Musculoskeletal system**	**2**	**0.80%**
Osteosarcoma	1	50.00%
Chondrosarcoma	1	50.00%
**Ocular system**	**3**	**1.20%**
Iridociliary adenocarcinoma	1	33.33%
Iridociliary adenoma	1	33.33%
Meibomian adenoma	1	33.33%
**Oral cavity**	**5**	**2.01%**
Melanoma	2	40.00%

Acanthomatous ameloblastoma	1	20.00%
Ameloblastic fibroma	1	20.00%
Peripheral odontogenic fibroma	1	20.00%
**German Shorthaired Pointer**	**23**	**0.36%**
**Cutaneous and soft tissue**	**10**	**43.48%**
Extraskeletal osteosarcoma	1	10.00%
Fibrosarcoma	1	10.00%
Hemangioma	1	10.00%
Hemangiosarcoma	1	10.00%
Lipoma	1	10.00%
Lymphoma	1	10.00%
Melanocytoma	1	10.00%
Melanoma	1	10.00%
Pilomatricoma	1	10.00%
Sebaceous epithelioma	1	10.00%
**Gastrointestinal tract**	**2**	**8.70%**
Intestinal adenoma	1	50.00%
Intestinal lymphoma	1	50.00%
**Haemolymphatic system**	**1**	**4.35%**
Splenic lymphoma	1	100.00%
**Male reproductive system**	**1**	**4.35%**
Seminoma	1	100.00%
**Mammary**	**6**	**26.09%**
Malignant mammary tumour	5	83.33%
Benign mammary tumour	1	16.67%
**Musculoskeletal system**	**1**	**4.35%**
Osteosarcoma	1	100.00%
**Ocular system**	**1**	**4.35%**
Iridociliary adenoma	1	100.00%
**Oral cavity**	**1**	**4.35%**
Peripheral odontogenic fibroma	1	100.00%
**Golden Retriever**	**179**	**2.81%**
**Cutaneous and soft tissue**	**106**	**59.22%**
Mast cell tumor (Grade II)	22	20.75%
Melanoma	8	7.55%
Lipoma	7	6.60%
Perivascular wall tumor	7	6.60%
Subcutaneous mast cell tumor	7	6.60%
Trichoepithelioma	6	5.66%
Hepatoid adenoma	5	4.72%
Mast cell tumor (Grade I)	5	4.72%
Mast cell tumor (Grade III)	5	4.72%
Squamous papilloma	5	4.72%
Fibrosarcoma	4	3.77%
Hemangiosarcoma	4	3.77%
Histiocytoma	4	3.77%
Squamous cell carcinoma	4	3.77%
Apocrine adenoma	3	2.83%

Hemangioma	2	1.89%
Myxosarcoma	2	1.89%
Anal sac adenocarcinoma	1	0.94%
Basosquamous carcinoma	1	0.94%
Lymphoma	1	0.94%
Melanocytoma	1	0.94%
Pilomatricoma	1	0.94%
Plasmacytoma	1	0.94%
**Female reproductive system**	**3**	**1.68%**
Benign ovarian tumor	3	100.00%
**Gastrointestinal tract**	**7**	**3.91%**
GIST	2	28.57%
Hepatocelular carcinoma	2	28.57%
Intestinal adenocarcinoma	2	28.57%
Intestinal lymphoma	1	14.29%
**Haemolymphatic system**	**6**	**3.35%**
Lymphoma	2	33.33%
Splenic hemangiosarcoma	2	33.33%
Splenic lymphoma	2	33.33%
**Male reproductive system**	**18**	**10.06%**
Intersticial cell tumor	10	55.56%
Seminoma	5	27.78%
Mixed germ cell sex-cord stromal tumor	2	11.11%
Sertoli cell tumor	1	5.56%
**Mammary**	**16**	**8.94%**
Malignant mammary tumour	14	87.50%
Benign mammary tumour	2	12.50%
**Musculoskeletal system**	**2**	**1.12%**
Chondrosarcoma	1	50.00%
Osteosarcoma	1	50.00%
**Ocular system**	**13**	**7.26%**
Meibomian epithelioma	8	61.54%
Meibomian adenoma	4	30.77%
Melanocytoma	1	7.69%
**Oral cavity**	**8**	**4.47%**
Peripheral odontogenic fibroma	5	62.50%
Melanoma	3	37.50%
**Grand Bleu de Gascogne**	**2**	**0.03%**
**Cutaneous and soft tissue**	**2**	**100.00%**
Histiocytoma	1	50.00%
Mast cell tumor (Grade I)	1	50.00%
**Great Dane**	**18**	**0.28%**
**Cutaneous and soft tissue**	**9**	**50.00%**
Histiocytoma	2	22.22%
Mast cell tumor (Grade II)	2	22.22%
Apocrine adenoma	1	11.11%
Hemangiosarcoma	1	11.11%
Mast cell tumor (Grade III)	1	11.11%

Pilomatricoma	1	11.11%
Plasmacytoma	1	11.11%
**Gastrointestinal tract**	**1**	**5.56%**
Hepatocelular carcinoma	1	100.00%
**Male reproductive system**	**1**	**5.56%**
Intersticial cell tumor	1	100.00%
**Mammary**	**1**	**5.56%**
Malignant mammary tumour	1	100.00%
**Musculoskeletal system**	**1**	**5.56%**
Osteosarcoma	1	100.00%
**Ocular system**	**3**	**16.67%**
Meibomian adenoma	3	100.00%
**Oral cavity**	**1**	**5.56%**
Peripheral odontogenic fibroma	1	100.00%
**Urinary system**	**1**	**5.56%**
Nefroblastoma	1	100.00%
**Greyhound**	**10**	**0.16%**
**Cutaneous and soft tissue**	**8**	**80.00%**
Perivascular wall tumor	2	25.00%
Squamous cell carcinoma	2	25.00%
Hemangioma	1	12.50%
Hemangiosarcoma	1	12.50%
Hepatoid adenoma	1	12.50%
Lipoma	1	12.50%
**Mammary**	**1**	**10.00%**
Malignant mammary tumour	1	100.00%
**Ocular system**	**1**	**10.00%**
Meibomian adenoma	1	100.00%
**Jack Russel**	**46**	**0.72%**
**Cutaneous and soft tissue**	**32**	**69.57%**
Histiocytoma	7	21.88%
Mast cell tumor (Grade II)	5	15.63%
Mast cell tumor (Grade I)	4	12.50%
Lipoma	3	9.38%
Subcutaneous mast cell tumor	3	9.38%
Hemangiosarcoma	2	6.25%
Apocrine adenocarcinoma	1	3.13%
Apocrine adenoma	1	3.13%
Basosquamous carcinoma	1	3.13%
Fibrosarcoma	1	3.13%
Hemangioma	1	3.13%
Hepatoid epithelioma	1	3.13%
Myxoma	1	3.13%
Sebaceous adenoma	1	3.13%
**Gastrointestinal tract**	**1**	**2.17%**
Intestinal adenoma	1	100.00%
**Male reproductive system**	**3**	**6.52%**
Intersticial cell tumor	2	66.67%

Mixed germ cell sex-cord stromal tumor	1	33.33%
**Mammary**	**6**	**13.04%**
Malignant mammary tumour	5	83.33%
Benign mammary tumour	1	16.67%
**Ocular system**	**1**	**2.17%**
Meibomian adenoma	1	100.00%
**Oral cavity**	**3**	**6.52%**
Peripheral odontogenic fibroma	3	100.00%
**Japanese Spitz**	**2**	**0.03%**
**Mammary**	**2**	**100.00%**
Benign mammary tumour	1	50.00%
Malignant mammary tumour	1	50.00%
**Labrador Retriever**	**729**	**11.46%**
**Cutaneous and soft tissue**	**482**	**66.12%**
Mast cell tumor (Grade II)	105	21.78%
Lipoma	52	10.79%
Subcutaneous mast cell tumor	37	7.68%
Mast cell tumor (Grade I)	28	5.81%
Mast cell tumor (Grade III)	23	4.77%
Fibrosarcoma	20	4.15%
Hepatoid adenoma	18	3.73%
Histiocytoma	18	3.73%
Melanoma	17	3.53%
Perivascular wall tumor	17	3.53%
Sebaceous epithelioma	16	3.32%
Hemangioma	13	2.70%
Hemangiosarcoma	11	2.28%
Squamous papilloma	10	2.07%
Trichoblastoma	10	2.07%
Fibroma	9	1.87%
Trichoepithelioma	9	1.87%
Squamous cell carcinoma	8	1.66%
Apocrine adenoma	6	1.24%
Melanocytoma	5	1.04%
Hemangiosarcoma	4	0.83%
Hepatoid epithelioma	4	0.83%
Pilomatricoma	4	0.83%
Plasmacytoma	4	0.83%
Schwannoma	4	0.83%
Apocrine adenocarcinoma	3	0.62%
Lymphoma	3	0.62%
Myxosarcoma	3	0.62%
Sebaceous adenoma	3	0.62%
Basal cell carcinoma	2	0.41%
Myxoma	2	0.41%
Apocrine ductal adenocarcinoma	1	0.21%
Basosquamous carcinoma	1	0.21%
Ceruminous adenocarcinoma	1	0.21%

Cutaneous histiocytosis	1	0.21%
Hepatoid adenocarcinoma	1	0.21%
Histiocytic sarcoma	1	0.21%
Infundibular keratinizing acanthoma	1	0.21%
Liposarcoma	1	0.21%
Lymphangioma	1	0.21%
Malignant trichoepithelioma	1	0.21%
Matrical carcinoma	1	0.21%
Mesothelioma	1	0.21%
Plasmacytoma	1	0.21%
Sebaceous adenocarcinoma	1	0.21%
**Female reproductive system**	**19**	**2.61%**
Benign ovarian tumor	9	47.37%
Leiomyoma	5	26.32%
Ovarian carcinoma	4	21.05%
Uterine carcinoma	1	5.26%
**Gastrointestinal tract**	**8**	**1.10%**
Intestinal adenocarcinoma	3	37.50%
Intestinal adenoma	2	25.00%
Cholangiocarcinoma	1	12.50%
GIST	1	12.50%
Hepatic lymphoma	1	12.50%
**Haemolymphatic system**	**13**	**1.78%**
Splenic hemangiosarcoma	6	46.15%
Splenic Fibrosarcoma	2	15.38%
Splenic lymphoma	2	15.38%
Lymphoma	1	7.69%
Splenic Leiomyosarcoma	1	7.69%
Splenic Myelolipoma	1	7.69%
**Male reproductive system**	**38**	**5.21%**
Intersticial cell tumor	20	52.63%
Sertoli cell tumor	9	23.68%
Mixed germ cell sex-cord stromal tumor	4	10.53%
Seminoma	4	10.53%
Seminoma	1	2.63%
**Mammary**	**98**	**13.44%**
Malignant mammary tumour	84	85.71%
Benign mammary tumour	14	14.29%
**Musculoskeletal system**	**6**	**0.82%**
Osteosarcoma	6	100.00%
**Neuroendocrine**	**2**	**0.27%**
Thyroid carcinoma	2	100.00%
**Ocular system**	**41**	**5.62%**
Meibomian adenoma	36	87.80%
Meibomian epithelioma	3	7.32%
Iridociliary adenoma	1	2.44%
Melanoma	1	2.44%
**Oral cavity**	**19**	**2.61%**

Peripheral odontogenic fibroma	9	47.37%
Melanoma	5	26.32%
Acanthomatous ameloblastoma	3	15.79%
Granular cell myoblastoma	1	5.26%
Malignant plasmacytoma	1	5.26%
**Respiratory System**	**1**	**0.14%**
Nasal adenocarcinoma	1	100.00%
**Urinary system**	**2**	**0.27%**
Renal adenocarcinoma	1	50.00%
Urothelial cell carcinoma	1	50.00%
**Lhasa Apso**	**5**	**0.08%**
**Cutaneous and soft tissue**	**1**	**20.00%**
Sebaceous adenoma	1	100.00%
**Mammary**	**3**	**60.00%**
Malignant mammary tumour	2	66.67%
Benign mammary tumour	1	33.33%
**Oral cavity**	**1**	**20.00%**
Melanoma	1	100.00%
**Maltese**	**25**	**0.39%**
**Cutaneous and soft tissue**	**14**	**56.00%**
Lipoma	2	14.29%
Squamous cell carcinoma	2	14.29%
Squamous papilloma	2	14.29%
Fibroma	1	7.14%
Mast cell tumor (Grade I)	1	7.14%
Myxosarcoma	1	7.14%
Perivascular wall tumor	1	7.14%
Sebaceous adenoma	1	7.14%
Sebaceous epithelioma	1	7.14%
Subcutaneous mast cell tumor	1	7.14%
Trichoblastoma	1	7.14%
**Female reproductive system**	**2**	**8.00%**
Benign ovarian tumor	1	50.00%
Leiomyoma	1	50.00%
**Mammary**	**9**	**36.00%**
Malignant mammary tumour	6	66.67%
Benign mammary tumour	3	33.33%
**Miniature Schnauzer**	**19**	**0.30%**
**Cutaneous and soft tissue**	**10**	**52.63%**
Lipoma	3	30.00%
Hepatoid adenoma	2	20.00%
Basosquamous carcinoma	1	10.00%
Melanoma	1	10.00%
Pilomatricoma	1	10.00%
Squamous papilloma	1	10.00%
Trichoepithelioma	1	10.00%
**Male reproductive system**	**1**	**5.26%**
Intersticial cell tumor	1	100.00%

**Mammary**	**4**	**21.05%**
Malignant mammary tumour	3	75.00%
Benign mammary tumour	1	25.00%
**Oral cavity**	**3**	**15.79%**
Melanoma	3	100.00%
**Urinary system**	**1**	**5.26%**
Urothelial cell carcinoma	1	100.00%
**Mixed-Breed**	**2660**	**41.83%**
**Cutaneous and soft tissue**	**1534**	**57.67%**
Perivascular wall tumor	195	12.71%
Lipoma	162	10.56%
Mast cell tumor (Grade II)	125	8.15%
Hepatoid adenoma	88	5.74%
Hemangiosarcoma	76	4.95%
Histiocytoma	70	4.56%
Mast cell tumor (Grade I)	60	3.91%
Fibrosarcoma	53	3.46%
Melanoma	49	3.19%
Mast cell tumor (Grade III)	46	3.00%
Trichoblastoma	45	2.93%
Hemangioma	44	2.87%
Squamous cell carcinoma	43	2.80%
Infundibular keratinizing acanthoma	40	2.61%
Melanocytoma	40	2.61%
Sebaceous epithelioma	33	2.15%
Subcutaneous mast cell tumor	33	2.15%
Hepatoid epithelioma	31	2.02%
Sebaceous adenoma	31	2.02%
Trichoepithelioma	30	1.96%
Schwannoma	29	1.89%
Apocrine adenoma	27	1.76%
Squamous papilloma	25	1.63%
Apocrine adenocarcinoma	18	1.17%
Fibroma	17	1.11%
Hemangiosarcoma	15	0.98%
Pilomatricoma	13	0.85%
Anal sac adenocarcinoma	11	0.72%
Plasmacytoma	10	0.65%
Lymphoma	8	0.52%
Malignant trichoepithelioma	7	0.46%
Matrical carcinoma	7	0.46%
Myxosarcoma	7	0.46%
Liposarcoma	6	0.39%
Tricholemmoma	6	0.39%
Hepatoid adenocarcinoma	5	0.33%
Apocrine ductal adenocarcinoma	4	0.26%
Histiocytic sarcoma	3	0.20%
Malignant plasmacytoma	3	0.20%

Ceruminous adenocarcinoma	2	0.13%
Clear cell adnexal carcinoma	2	0.13%
Malignant schwanoma	2	0.13%
Plasmacytoma	2	0.13%
Extraskeletal chondroma	1	0.07%
Extraskeletal chondrosarcoma	1	0.07%
Hepatoid carcinoma	1	0.07%
Inverted squamous papilloma	1	0.07%
Leiomyoma	1	0.07%
Liposarcoma	1	0.07%
Lymphangioma	1	0.07%
Lymphangiosarcoma	1	0.07%
Malignant plasmacytoma	1	0.07%
Merkel cell tumor	1	0.07%
Sebaceous adenocarcinoma	1	0.07%
**Female reproductive system**	**50**	**1.88%**
Leiomyoma	31	62.00%
Benign ovarian tumor	12	24.00%
Ovarian carcinoma	4	8.00%
Dysgerminoma	3	6.00%
**Gastrointestinal tract**	**48**	**1.80%**
Intestinal adenocarcinoma	12	25.00%
Hepatic Hemangiosarcoma	7	14.58%
Hepatocelular carcinoma	7	14.58%
Intestinal lymphoma	6	12.50%
Intestinal adenoma	4	8.33%
GIST	3	6.25%
Salivary gland carcinoma	3	6.25%
Leiomyosarcoma	2	4.17%
Biliary adenoma	1	2.08%
Extraskeletal osteosarcoma	1	2.08%
Hepatocelular adenoma	1	2.08%
Signet-ring cell carcinoma	1	2.08%
**Haemolymphatic system**	**62**	**2.33%**
Splenic hemangiosarcoma	27	43.55%
Lymphoma	19	30.65%
Splenic lymphoma	6	9.68%
Splenic Fibrosarcoma	3	4.84%
Splenic Myelolipoma	3	4.84%
Splenic Anaplasic sarcoma	2	3.23%
Splenic Plasmacytoma	1	1.61%
Thymic lymphoma	1	1.61%
**Male reproductive system**	**132**	**4.96%**
Intersticial cell tumor	54	40.91%
Sertoli cell tumor	31	23.48%
Seminoma	29	21.97%
Mixed germ cell sex-cord stromal tumor	14	10.61%
Seminoma	3	2.27%

TVT	1	0.76%
**Mammary**	**664**	**24.96%**
Malignant mammary tumour	544	81.93%
Benign mammary tumour	120	18.07%
**Musculoskeletal system**	**18**	**0.68%**
Osteosarcoma	17	94.44%
Chondrosarcoma	1	5.56%
**Neuroendocrine**	**8**	**0.30%**
Thyroid carcinoma	5	62.50%
Chemodectoma	1	12.50%
Insulinoma	1	12.50%
Pheochromocytoma	1	12.50%
**Ocular system**	**67**	**2.52%**
Meibomian adenoma	49	73.13%
Meibomian epithelioma	11	16.42%
Melanocytoma	3	4.48%
Iridociliary adenoma	2	2.99%
Melanoma	2	2.99%
**Oral cavity**	**61**	**2.29%**
Peripheral odontogenic fibroma	31	50.82%
Melanoma	14	22.95%
Acanthomatous ameloblastoma	9	14.75%
Fibrosarcoma	3	4.92%
Granular cell myoblastoma	2	3.28%
Ameloblastic fibroma	1	1.64%
Plasmacytoma	1	1.64%
**Respiratory System**	**4**	**0.15%**
Pulmonary adenocarcinoma	2	50.00%
Nasal adenocarcinoma	1	25.00%
Nasal transitional cell carcinoma	1	25.00%
**Urinary system**	**12**	**0.45%**
Urothelial cell carcinoma	6	50.00%
Renal adenocarcinoma	3	25.00%
Renal adenoma	1	8.33%
Renal fibrosarcoma	1	8.33%
Renal hemangioma	1	8.33%
**Old English Sheepdog**	**1**	**0.02%**
**Cutaneous and soft tissue**	**1**	**100.00%**
Hepatoid epithelioma	1	100.00%
**Papillon**	**2**	**0.03%**
**Mammary**	**1**	**50.00%**
Malignant mammary tumour	1	100.00%
**Ocular system**	**1**	**50.00%**
Meibomian adenoma	1	100.00%
**Pekingese**	**41**	**0.64%**
**Cutaneous and soft tissue**	**17**	**41.46%**
Hemangioma	2	11.76%
Melanocytoma	2	11.76%

Myxosarcoma	2	11.76%
Schwannoma	2	11.76%
Squamous cell carcinoma	2	11.76%
Ceruminous adenoma	1	5.88%
Hemangiosarcoma	1	5.88%
Histiocytoma	1	5.88%
Infundibular keratinizing acanthoma	1	5.88%
Leiomyosarcoma	1	5.88%
Sebaceous adenoma	1	5.88%
Trichoblastoma	1	5.88%
**Female reproductive system**	**1**	**2.44%**
Leiomyoma	1	100.00%
**Gastrointestinal tract**	**1**	**2.44%**
Intestinal lymphoma	1	100.00%
**Haemolymphatic system**	**1**	**2.44%**
Lymphoma	1	100.00%
**Male reproductive system**	**3**	**7.32%**
Intersticial cell tumor	2	66.67%
Mixed germ cell sex-cord stromal tumor	1	33.33%
**Mammary**	**15**	**36.59%**
Malignant mammary tumour	11	73.33%
Benign mammary tumour	4	26.67%
**Ocular system**	**3**	**7.32%**
Meibomian adenoma	3	100.00%
**Pinscher**	**161**	**2.53%**
**Cutaneous and soft tissue**	**67**	**41.61%**
Mast cell tumor (Grade II)	9	13.43%
Melanocytoma	8	11.94%
Histiocytoma	6	8.96%
Melanoma	5	7.46%
Infundibular keratinizing acanthoma	4	5.97%
Mast cell tumor (Grade III)	4	5.97%
Squamous cell carcinoma	4	5.97%
Hemangioma	3	4.48%
Fibrosarcoma	2	2.99%
Hemangiosarcoma	2	2.99%
Hepatoid adenoma	2	2.99%
Hepatoid epithelioma	2	2.99%
Lipoma	2	2.99%
Mast cell tumor (Grade I)	2	2.99%
Perivascular wall tumor	2	2.99%
Schwannoma	2	2.99%
Subcutaneous mast cell tumor	2	2.99%
Apocrine ductal adenocarcinoma	1	1.49%
Fibroma	1	1.49%
Matrical carcinoma	1	1.49%
Plasmacytoma	1	1.49%
Sebaceous adenoma	1	1.49%

Squamous papilloma	1	1.49%
**Female reproductive system**	**3**	**1.86%**
Dysgerminoma	1	33.33%
Leiomyoma	1	33.33%
Ovarian carcinoma	1	33.33%
**Gastrointestinal tract**	**1**	**0.62%**
Intestinal adenocarcinoma	1	100.00%
**Haemolymphatic system**	**1**	**0.62%**
Splenic Fibrosarcoma	1	100.00%
**Male reproductive system**	**11**	**6.83%**
Sertoli cell tumor	4	36.36%
Seminoma	3	27.27%
Intersticial cell tumor	2	18.18%
Mixed germ cell sex-cord stromal tumor	2	18.18%
**Mammary**	**72**	**44.72%**
Malignant mammary tumour	47	65.28%
Benign mammary tumour	25	34.72%
**Neuroendocrine**	**1**	**0.62%**
Thyroid carcinoma	1	100.00%
**Oral cavity**	**4**	**2.48%**
Peripheral odontogenic fibroma	4	100.00%
**Urinary system**	**1**	**0.62%**
Urothelial papiloma	1	100.00%
**Pomeranian**	**7**	**0.11%**
**Cutaneous and soft tissue**	**3**	**42.86%**
Perivascular wall tumor	1	33.33%
Squamous cell carcinoma	1	33.33%
Trichoblastoma	1	33.33%
**Male reproductive system**	**1**	**14.29%**
Intersticial cell tumor	1	100.00%
**Mammary**	**1**	**14.29%**
Malignant mammary tumour	1	100.00%
**Oral cavity**	**2**	**28.57%**
Melanoma	1	50.00%
Peripheral odontogenic fibroma	1	50.00%
**Poodle**	**166**	**2.61%**
**Cutaneous and soft tissue**	**55**	**33.13%**
Lipoma	7	12.73%
Hepatoid adenoma	5	9.09%
Infundibular keratinizing acanthoma	5	9.09%
Sebaceous adenoma	5	9.09%
Trichoblastoma	4	7.27%
Hepatoid epithelioma	2	3.64%
Mast cell tumor (Grade I)	2	3.64%
Mast cell tumor (Grade II)	2	3.64%
Melanoma	2	3.64%
Perivascular wall tumor	2	3.64%
Plasmacytoma	2	3.64%

Sebaceous epithelioma	2	3.64%
Trichoepithelioma	2	3.64%
Anal sac adenocarcinoma	1	1.82%
Apocrine adenocarcinoma	1	1.82%
Apocrine ductal adenocarcinoma	1	1.82%
Ceruminous adenoma	1	1.82%
Fibroma	1	1.82%
Fibrosarcoma	1	1.82%
Hemangioma	1	1.82%
Malignant schwanoma	1	1.82%
Matrical carcinoma	1	1.82%
Pilomatricoma	1	1.82%
Squamous cell carcinoma	1	1.82%
Squamous papilloma	1	1.82%
Subcutaneous mast cell tumor	1	1.82%
**Female reproductive system**	**2**	**1.20%**
Leiomyoma	2	100.00%
**Gastrointestinal tract**	**1**	**0.60%**
GIST	1	100.00%
**Haemolymphatic system**	**5**	**3.01%**
Lymphoma	3	60.00%
Splenic hemangiosarcoma	1	20.00%
Splenic lymphoma	1	20.00%
**Male reproductive system**	**4**	**2.41%**
Intersticial cell tumor	2	50.00%
Seminoma	2	50.00%
**Mammary**	**94**	**56.63%**
Malignant mammary tumour	78	82.98%
Benign mammary tumour	16	17.02%
**Neuroendocrine**	**1**	**0.60%**
Adrenocortical carcinoma	1	100.00%
**Ocular system**	**4**	**2.41%**
Meibomian adenoma	2	50.00%
Meibomian epithelioma	1	25.00%
Melanoma	1	25.00%
**Portuguese Podengo**	**62**	**0.97%**
**Cutaneous and soft tissue**	**34**	**54.84%**
Mast cell tumor (Grade II)	7	20.59%
Perivascular wall tumor	4	11.76%
Hepatoid adenoma	3	8.82%
Sebaceous adenoma	3	8.82%
Fibrosarcoma	2	5.88%
Histiocytoma	2	5.88%
Lipoma	2	5.88%
Melanoma	2	5.88%
Myxosarcoma	2	5.88%
Fibroma	1	2.94%
Hemangioma	1	2.94%

Hepatoid epithelioma	1	2.94%
Infundibular keratinizing acanthoma	1	2.94%
Lymphoma	1	2.94%
Squamous cell carcinoma	1	2.94%
Trichoblastoma	1	2.94%
**Gastrointestinal tract**	**1**	**1.61%**
Intestinal adenocarcinoma	1	100.00%
**Haemolymphatic system**	**2**	**3.23%**
Lymphoma	2	100.00%
**Male reproductive system**	**1**	**1.61%**
Sertoli cell tumor	1	100.00%
**Mammary**	**19**	**30.65%**
Malignant mammary tumour	17	89.47%
Benign mammary tumour	2	10.53%
**Musculoskeletal system**	**1**	**1.61%**
Osteosarcoma	1	100.00%
**Ocular system**	**3**	**4.84%**
Meibomian adenoma	2	66.67%
Meibomian epithelioma	1	33.33%
**Urinary system**	**1**	**1.61%**
Urothelial cell carcinoma	1	100.00%
**Portuguese Sheepdog**	**1**	**0.02%**
**Mammary**	**1**	**100.00%**
Benign mammary tumour	1	100.00%
**Portuguese Water Dog**	**22**	**0.35%**
**Cutaneous and soft tissue**	**12**	**54.55%**
Lipoma	2	16.67%
Trichoblastoma	2	16.67%
Basosquamous carcinoma	1	8.33%
Infundibular keratinizing acanthoma	1	8.33%
Mast cell tumor (Grade II)	1	8.33%
Melanoma	1	8.33%
Perivascular wall tumor	1	8.33%
Sebaceous adenoma	1	8.33%
Squamous cell carcinoma	1	8.33%
Squamous papilloma	1	8.33%
**Gastrointestinal tract**	**1**	**4.55%**
Intestinal lymphoma	1	100.00%
**Haemolymphatic system**	**2**	**9.09%**
Lymphoma	2	100.00%
**Male reproductive system**	**3**	**13.64%**
Intersticial cell tumor	2	66.67%
Mixed germ cell sex-cord stromal tumor	1	33.33%
**Mammary**	**3**	**13.64%**
Malignant mammary tumour	3	100.00%
**Ocular system**	**1**	**4.55%**
Meibomian adenoma	1	100.00%
**Pug**	**32**	**0.50%**

**Cutaneous and soft tissue**	**27**	**84.38%**
Mast cell tumor (Grade II)	9	33.33%
Mast cell tumor (Grade I)	5	18.52%
Subcutaneous mast cell tumor	5	18.52%
Melanocytoma	2	7.41%
Histiocytoma	1	3.70%
Lymphoma	1	3.70%
Mast cell tumor (Grade III)	1	3.70%
Melanoma	1	3.70%
Plasmacytoma	1	3.70%
Trichoepithelioma	1	3.70%
**Haemolymphatic system**	**3**	**9.38%**
Lymphoma	3	100.00%
**Male reproductive system**	**1**	**3.13%**
Seminoma	1	100.00%
**Ocular system**	**1**	**3.13%**
Meibomian adenoma	1	100.00%
**Pyrenean Mastiff**	**1**	**0.02%**
**Cutaneous and soft tissue**	**1**	**100.00%**
Hemangiosarcoma	1	100.00%
**Pyrenean Mountain Dog**	**1**	**0.02%**
**Cutaneous and soft tissue**	**1**	**100.00%**
Hemangiosarcoma	1	100.00%
**Ratonero bodeguero**	**1**	**0.02%**
**Male reproductive system**	**1**	**100.00%**
Mixed germ cell sex-cord stromal tumor	1	100.00%
**Rhodesian Ridgeback**	**20**	**0.31%**
**Cutaneous and soft tissue**	**14**	**70.00%**
Mast cell tumor (Grade I)	2	14.29%
Sebaceous epithelioma	2	14.29%
Trichoepithelioma	2	14.29%
Ceruminous adenocarcinoma	1	7.14%
Hemangiosarcoma	1	7.14%
Hepatoid adenoma	1	7.14%
Mast cell tumor (Grade II)	1	7.14%
Melanocytoma	1	7.14%
Melanoma	1	7.14%
Perivascular wall tumor	1	7.14%
Subcutaneous mast cell tumor	1	7.14%
**Male reproductive system**	**1**	**5.00%**
Intersticial cell tumor	1	100.00%
**Mammary**	**5**	**25.00%**
Malignant mammary tumour	5	100.00%
**Rottweiler**	**20**	**0.31%**
**Cutaneous and soft tissue**	**13**	**65.00%**
Fibrosarcoma	2	15.38%
Lipoma	2	15.38%
Melanoma	2	15.38%

Extraskeletal osteosarcoma	1	7.69%
Hemangiosarcoma	1	7.69%
Histiocytoma	1	7.69%
Infundibular keratinizing acanthoma	1	7.69%
Mast cell tumor (Grade III)	1	7.69%
Melanocytoma	1	7.69%
Squamous cell carcinoma	1	7.69%
**Gastrointestinal tract**	**1**	**5.00%**
Intestinal adenocarcinoma	1	100.00%
**Haemolymphatic system**	**2**	**10.00%**
Splenic hemangiosarcoma	2	100.00%
**Male reproductive system**	**1**	**5.00%**
Seminoma	1	100.00%
**Mammary**	**1**	**5.00%**
Malignant mammary tumour	1	100.00%
**Oral cavity**	**2**	**10.00%**
Fibrosarcoma	1	50.00%
Melanoma	1	50.00%
**Rough Collie**	**1**	**0.02%**
**Ocular system**	**1**	**100.00%**
Meibomian adenoma	1	100.00%
**Samoyed**	**8**	**0.13%**
**Cutaneous and soft tissue**	**3**	**37.50%**
Mast cell tumor (Grade III)	1	33.33%
Sebaceous adenoma	1	33.33%
Trichoblastoma	1	33.33%
**Male reproductive system**	**1**	**12.50%**
Intersticial cell tumor	1	100.00%
**Mammary**	**4**	**50.00%**
Malignant mammary tumour	4	100.00%
**Saint Miguel Cattle Dog**	**12**	**0.19%**
**Cutaneous and soft tissue**	**7**	**58.33%**
Trichoepithelioma	2	28.57%
Hepatoid adenoma	1	14.29%
Histiocytoma	1	14.29%
Malignant trichoepithelioma	1	14.29%
Melanoma	1	14.29%
Pilomatricoma	1	14.29%
**Gastrointestinal tract**	**1**	**8.33%**
Cholangiocarcinoma	1	100.00%
**Mammary**	**2**	**16.67%**
Malignant mammary tumour	2	100.00%
**Musculoskeletal system**	**1**	**8.33%**
Osteosarcoma	1	100.00%
**Oral cavity**	**1**	**8.33%**
Granular cell myoblastoma	1	100.00%
**Saint Bernard**	**6**	**0.09%**
**Cutaneous and soft tissue**	**4**	**66.67%**

Apocrine adenocarcinoma	2	50.00%
Hemangioma	1	25.00%
Trichoblastoma	1	25.00%
**Musculoskeletal system**	**1**	**16.67%**
Osteosarcoma	1	100.00%
**Ocular system**	**1**	**16.67%**
Meibomian adenoma	1	100.00%
**Scottish Terrier**	**2**	**0.03%**
**Cutaneous and soft tissue**	**1**	**50.00%**
Histiocytoma	1	100.00%
**Mammary**	**1**	**50.00%**
Benign mammary tumour	1	100.00%
**Shar-Pei**	**33**	**0.52%**
**Cutaneous and soft tissue**	**28**	**84.85%**
Hemangioma	4	14.29%
Mast cell tumor (Grade II)	4	14.29%
Perivascular wall tumor	4	14.29%
Histiocytoma	3	10.71%
Melanoma	3	10.71%
Mast cell tumor (Grade III)	2	7.14%
Apocrine adenoma	1	3.57%
Cutaneous histiocytosis	1	3.57%
Fibrosarcoma	1	3.57%
Lipoma	1	3.57%
Lymphangioma	1	3.57%
Mast cell tumor (Grade I)	1	3.57%
Melanocytoma	1	3.57%
Trichoblastoma	1	3.57%
**Gastrointestinal tract**	**1**	**3.03%**
Intestinal lymphoma	1	100.00%
**Haemolymphatic system**	**1**	**3.03%**
Lymphoma	1	100.00%
**Male reproductive system**	**1**	**3.03%**
Intersticial cell tumor	1	100.00%
**Mammary**	**2**	**6.06%**
Benign mammary tumour	1	50.00%
Malignant mammary tumour	1	50.00%
**Shihtzu**	**44**	**0.69%**
**Cutaneous and soft tissue**	**21**	**47.73%**
Basal cell carcinoma	2	9.52%
Hepatoid epithelioma	2	9.52%
Lipoma	2	9.52%
Perivascular wall tumor	2	9.52%
Sebaceous epithelioma	2	9.52%
Squamous papilloma	2	9.52%
Apocrine adenocarcinoma	1	4.76%
Apocrine adenoma	1	4.76%
Ceruminous adenocarcinoma	1	4.76%

Hepatoid adenoma	1	4.76%
Infundibular keratinizing acanthoma	1	4.76%
Pilomatricoma	1	4.76%
Sebaceous adenoma	1	4.76%
Trichoblastoma	1	4.76%
Trichoepithelioma	1	4.76%
**Haemolymphatic system**	**1**	**2.27%**
Lymphoma	1	100.00%
**Male reproductive system**	**1**	**2.27%**
Seminoma	1	100.00%
**Mammary**	**19**	**43.18%**
Malignant mammary tumour	12	63.16%
Benign mammary tumour	7	36.84%
**Ocular system**	**1**	**2.27%**
Meibomian adenoma	1	100.00%
**Oral cavity**	**1**	**2.27%**
Peripheral odontogenic fibroma	1	100.00%
**Siberian Husky**	**22**	**0.35%**
**Cutaneous and soft tissue**	**13**	**59.09%**
Perivascular wall tumor	5	38.46%
Hepatoid adenoma	2	15.38%
Apocrine adenoma	1	7.69%
Ceruminous adenocarcinoma	1	7.69%
Hemangioma	1	7.69%
Histiocytoma	1	7.69%
Malignant plasmacytoma	1	7.69%
Sebaceous epithelioma	1	7.69%
**Gastrointestinal tract**	**1**	**4.55%**
Intestinal lymphoma	1	100.00%
**Haemolymphatic system**	**1**	**4.55%**
Splenic Anaplasic sarcoma	1	100.00%
**Male reproductive system**	**1**	**4.55%**
Seminoma	1	100.00%
**Mammary**	**3**	**13.64%**
Benign mammary tumour	2	66.67%
Malignant mammary tumour	1	33.33%
**Musculoskeletal system**	**1**	**4.55%**
Chondrosarcoma	1	100.00%
**Ocular system**	**1**	**4.55%**
Meibomian adenoma	1	100.00%
**Urinary system**	**1**	**4.55%**
Urothelial cell carcinoma	1	100.00%
**Spanish Greyhound**	**1**	**0.02%**
**Mammary**	**1**	**100.00%**
Malignant mammary tumour	1	100.00%
**Spitz**	**20**	**0.31%**
**Cutaneous and soft tissue**	**8**	**40.00%**
Fibroma	1	12.50%

Hemangioma	1	12.50%
Hepatoid adenoma	1	12.50%
Myxosarcoma	1	12.50%
Perivascular wall tumor	1	12.50%
Plasmacytoma	1	12.50%
Sebaceous epithelioma	1	12.50%
Squamous papilloma	1	12.50%
**Male reproductive system**	**3**	**15.00%**
Sertoli cell tumor	2	66.67%
Intersticial cell tumor	1	33.33%
**Mammary**	**9**	**45.00%**
Malignant mammary tumour	8	88.89%
Benign mammary tumour	1	11.11%
**Staffordshire Terrier**	**13**	**0.20%**
**Cutaneous and soft tissue**	**11**	**84.62%**
Hemangiosarcoma	2	18.18%
Apocrine adenocarcinoma	1	9.09%
Fibrosarcoma	1	9.09%
Hemangiosarcoma	1	9.09%
Hepatoid adenoma	1	9.09%
Histiocytoma	1	9.09%
Lipoma	1	9.09%
Melanocytoma	1	9.09%
Perivascular wall tumor	1	9.09%
Subcutaneous mast cell tumor	1	9.09%
**Mammary**	**2**	**15.38%**
Benign mammary tumour	1	50.00%
Malignant mammary tumour	1	50.00%
**Teckel**	**29**	**0.46%**
**Cutaneous and soft tissue**	**13**	**44.83%**
Fibroma	2	15.38%
Histiocytoma	2	15.38%
Mast cell tumor (Grade II)	2	15.38%
Squamous papilloma	2	15.38%
Hemangiosarcoma	1	7.69%
Lipoma	1	7.69%
Mast cell tumor (Grade I)	1	7.69%
Mast cell tumor (Grade III)	1	7.69%
Melanoma	1	7.69%
**Haemolymphatic system**	**1**	**3.45%**
Splenic hemangiosarcoma	1	100.00%
**Male reproductive system**	**2**	**6.90%**
Intersticial cell tumor	1	50.00%
Mixed germ cell sex-cord stromal tumor	1	50.00%
**Mammary**	**11**	**37.93%**
Malignant mammary tumour	11	100.00%
**Neuroendocrine**	**2**	**6.90%**
Thyroid carcinoma	1	50.00%

Chemodectoma	1	50.00%
**Terrier**	**1**	**0.02%**
**Cutaneous and soft tissue**	**1**	**100.00%**
Sebaceous adenoma	1	100.00%
**Tibetan Mastiff**	**1**	**0.02%**
**Mammary**	**1**	**100.00%**
Malignant mammary tumour	1	100.00%
**Toy Poodle**	**5**	**0.08%**
**Cutaneous and soft tissue**	**3**	**60.00%**
Hemangiosarcoma	1	33.33%
Lipoma	1	33.33%
Sebaceous epithelioma	1	33.33%
**Male reproductive system**	**1**	**20.00%**
Seminoma	1	100.00%
**Mammary**	**1**	**20.00%**
Malignant mammary tumour	1	100.00%
**Transmontano Mastiff**	**5**	**0.08%**
**Cutaneous and soft tissue**	**2**	**40.00%**
Hepatoid adenoma	1	50.00%
Trichoepithelioma	1	50.00%
**Mammary**	**3**	**60.00%**
Malignant mammary tumour	3	100.00%
**Weimaraner**	**20**	**0.31%**
**Cutaneous and soft tissue**	**17**	**85.00%**
Lipoma	5	29.41%
Apocrine adenoma	2	11.76%
Histiocytoma	2	11.76%
Apocrine ductal adenocarcinoma	1	5.88%
Hemangioma	1	5.88%
Malignant trichoepithelioma	1	5.88%
Mast cell tumor (Grade I)	1	5.88%
Perivascular wall tumor	1	5.88%
Plasmacytoma	1	5.88%
Subcutaneous mast cell tumor	1	5.88%
Trichoepithelioma	1	5.88%
**Female reproductive system**	**1**	**5.00%**
Benign ovarian tumor	1	100.00%
**Haemolymphatic system**	**1**	**5.00%**
Splenic hemangiosarcoma	1	100.00%
**Ocular system**	**1**	**5.00%**
Meibomian adenoma	1	100.00%
**West Highland White Terrier**	**7**	**0.11%**
**Cutaneous and soft tissue**	**3**	**42.86%**
Lipoma	1	33.33%
Subcutaneous mast cell tumor	1	33.33%
Trichoblastoma	1	33.33%
**Male reproductive system**	**1**	**14.29%**
Sertoli cell tumor	1	100.00%

**Mammary**	**3**	**42.86%**
Malignant mammary tumour	3	100.00%
**Whippet**	**1**	**0.02%**
**Cutaneous and soft tissue**	**1**	**100.00%**
Extraskeletal chondrosarcoma	1	100.00%
**White Swiss Shepherd Dog**	**6**	**0.09%**
**Cutaneous and soft tissue**	**3**	**50.00%**
Infundibular keratinizing acanthoma	1	33.33%
Schwannoma	1	33.33%
Sebaceous epithelioma	1	33.33%
**Mammary**	**2**	**33.33%**
Malignant mammary tumour	2	100.00%
**Respiratory System**	**1**	**16.67%**
Pulmonary adenocarcinoma	1	100.00%
**Yorkshire Terrier**	**274**	**4.31%**
**Cutaneous and soft tissue**	**94**	**34.31%**
Infundibular keratinizing acanthoma	13	13.83%
Histiocytoma	8	8.51%
Mast cell tumor (Grade II)	8	8.51%
Plasmacytoma	8	8.51%
Melanocytoma	6	6.38%
Sebaceous adenoma	5	5.32%
Mast cell tumor (Grade III)	4	4.26%
Perivascular wall tumor	4	4.26%
Trichoblastoma	4	4.26%
Fibrosarcoma	3	3.19%
Hepatoid epithelioma	3	3.19%
Melanoma	3	3.19%
Hemangioma	2	2.13%
Inverted squamous papilloma	2	2.13%
Lipoma	2	2.13%
Squamous cell carcinoma	2	2.13%
Subcutaneous mast cell tumor	2	2.13%
Apocrine adenocarcinoma	1	1.06%
Apocrine ductal adenocarcinoma	1	1.06%
Basal cell carcinoma	1	1.06%
Extraskeletal chondrosarcoma	1	1.06%
Fibroma	1	1.06%
Hepatoid adenoma	1	1.06%
Lymphoma	1	1.06%
Mast cell tumor (Grade I)	1	1.06%
Matrical carcinoma	1	1.06%
Pilomatricoma	1	1.06%
Plasmacytoma	1	1.06%
Schwannoma	1	1.06%
Sebaceous epithelioma	1	1.06%
Squamous papilloma	1	1.06%
Trichoepithelioma	1	1.06%

**Female reproductive system**	**3**	**1.09%**
Ovarian carcinoma	3	100.00%
**Gastrointestinal tract**	**2**	**0.73%**
GIST	1	50.00%
Plasmacytoma	1	50.00%
**Haemolymphatic system**	**1**	**0.36%**
Lymphoma	1	100.00%
**Male reproductive system**	**18**	**6.57%**
Intersticial cell tumor	9	50.00%
Seminoma	4	22.22%
Sertoli cell tumor	4	22.22%
Mixed germ cell sex-cord stromal tumor	1	5.56%
**Mammary**	**147**	**53.65%**
Malignant mammary tumour	100	68.03%
Benign mammary tumour	47	31.97%
**Ocular system**	**3**	**1.09%**
Meibomian adenoma	1	33.33%
Meibomian epithelioma	1	33.33%
Melanoma	1	33.33%
**Oral cavity**	**4**	**1.46%**
Peripheral odontogenic fibroma	3	75.00%
Melanoma	1	25.00%
**Urinary system**	**2**	**0.73%**
Renal lymphoma	1	50.00%
Vesical leiomyoma	1	50.00%
**Total**	**6359**	**100.00%**

GIST, Gastrointestinal Stromal Tumor
